# Plasmacytoid Dendritic Cells and the Control of Herpesvirus Infections

**DOI:** 10.3390/v1030383

**Published:** 2009-10-14

**Authors:** Thomas Baranek, Nicolas Zucchini, Marc Dalod

**Affiliations:** 1 Université de la Méditerranée, Centre d’Immunologie de Marseille-Luminy, Parc Scientifique & Technologique de Luminy, Case 906, F13288 Marseille, Cedex 09, France; E-Mails: baranek@ciml.univ-mrs.fr (T.B.); zucchini@ciml.univ-mrs.fr (N.Z.); 2 Institut National de la Santé et de la Recherche Médicale (INSERM), U631, Centre d’Immunologie de Marseille-Luminy, Parc Scientifique & Technologique de Luminy, Case 906, F13288 Marseille, Cedex 09, France; 3 Centre National de la Recherche Scientifique (CNRS), UMR6102, Centre d’Immunologie de Marseille-Luminy, Parc Scientifique & Technologique de Luminy, Case 906, F13288 Marseille, Cedex 09, France

**Keywords:** plasmacytoid dendritic cell, type-I interferon, mouse cytomegalovirus, human cytomegalovirus, herpesvirus type 1, herpesvirus type 2, immunotherapy

## Abstract

Type-I interferons (IFN-I) are cytokines essential for vertebrate antiviral defense, including against herpesviruses. IFN-I have potent direct antiviral activities and also mediate a multiplicity of immunoregulatory functions, which can either promote or dampen antiviral adaptive immune responses. Plasmacytoid dendritic cells (pDCs) are the professional producers of IFN-I in response to many viruses, including all of the herpesviruses tested. There is strong evidence that pDCs could play a major role in the initial orchestration of both innate and adaptive antiviral immune responses. Depending on their activation pattern, pDC responses may be either protective or detrimental to the host. Here, we summarize and discuss current knowledge regarding pDC implication in the physiopathology of mouse and human herpesvirus infections, and we discuss how pDC functions could be manipulated in immunotherapeutic settings to promote health over disease.

## 1. Introduction

Herpesviruses are enveloped viruses, with genomes consisting in double-stranded linear DNA. Nine herpesviruses have currently been associated with diseases in humans (Table 1). Three herpesviruses have been identified in mice. Herpesviruses establish life-long persistent infections in their natural hosts, with a very high seroprevalence ranging from 30% to 90% depending on the country and on the virus. The infection is usually asymptomatic in immunocompetent individuals. Upon resolution of the acute phase of the primary infection, herpesviruses establish latent infection in specific cell types. A variety of stress conditions, including immunosuppression or inflammation, promote herpesvirus reactivation which can eventually lead to severe pathology (for review see [1]). Indeed, herpesviruses are some of the most common opportunistic agents encountered in AIDS patients and in recipients of bone marrow or solid organ transplantation. Herpesviruses harbor a very selective tropism for their host species, attesting to a very long history of co-evolution spanning several millions of years. The mouse cytomegalovirus (MCMV) cannot infect rats or humans, and reciprocally for the human or rat CMVs. The genome of herpesviruses is of considerable size and complexity as compared to that of many other viruses. Herpesviruses harbor a high number of genes that are not essential for their replicative cycle but that appear to have been selected for escape from, or hijacking of, the host immune system. However, reciprocally, the immune systems of the hosts have evolved counterstrategies to control the viruses. One of the earliest and most potent responses of the hosts to infections with herpesviruses is the production of type-I interferons (IFN-I).

IFN-I were the first cytokines discovered, a little over 50 years ago, based on their direct, potent and broad antiviral activity [14,15]. More generally, IFN-I are now known to play an essential role in the global orchestration of antiviral immunity, by linking innate and adaptive immunity through multiple immunoregulatory functions [16]. For instance, IFN-I do not only play a crucial role in the control of the replication of many viruses, but they can also promote NK cell or CD8 T cell antiviral cytotoxic activity, either directly [17–20] or through the licensing of accessory cells such as conventional dendritic cells (cDCs) [13,21–24]. In mammals, there are one IFN-β and more than ten different IFN-α, the exact number of which varies across species. All of these IFN-I exert their activities by engaging an ubiquitous receptor (IFNAR) composed of two chains (IFNAR1 and IFNAR2) [25]. Although any cell type could theoretically produce IFN-I (at least IFN-β) when infected by a virus, one cell type specialized in the production of high levels of multiple species of IFN-I in response to herpesviruses and many other viruses has been identified and characterized in the last decade. It has been named IFN-I producing cells (IPCs) or plasmacytoid dendritic cells (pDCs).

In humans, pDCs are characterized by selective high level expression of the C-type lectin CLEC4C (also referred to as BDCA2) and of the immunoglobulin superfamily member LILRA4 (also referred to as ILT7). In mice, there are no orthologs of CLEC4C or LILRA4. Instead, mouse pDCs selectively express high levels of the C-type lectin SIGLECH and of the bone marrow stromal cell antigen 2 (BST2/CD317) membrane marker. SIGLECH is also found on a subset of marginal zone macrophages in the spleen. BST2 is induced at low to intermediate levels on a variety of immune cell types upon stimulation with IFN-I, including cDCs, and is also constitutively expressed on plasma cells. Therefore, mouse pDCs can be rigorously identified as CD11c^int^BST2^high^ or as CD11b^−^SIGLECH^+^. Although it has been frequently used, it is important to be aware that the combined expression of B220 and CD11c is not adequate to identify mouse pDCs, as B220^+^CD11c^+^ cells also encompass other leukocyte populations including a subset of NK cells [26–29] and a precursor of cDCs [30]. While mouse and human pDCs are identified by different markers, they share many functional characteristics [31] and selective expression of over 200 genes as compared to other leukocyte subsets [28]. Thus, there clearly is a very strong homology between mouse and human pDCs, such that the investigation of the functions of pDCs in vivo in the mouse model should be largely relevant for understanding their biology in humans. The initial discovery and the functional study of pDCs have been closely linked to the study of innate immune recognition of herpesviruses. Indeed, together with the influenza virus [32–34], herpes simplex viruses were the very first viruses used to identify human [2] or mouse [35] pDCs in vitro, based on the much older observation that a rare cell type had the unique ability to rapidly recognize herpesviruses for consecutive high level production of IFN-I [36,37]. We reported the first evidence that pDCs actually constitute the major source of systemic IFN-I production during a viral infection in vivo using MCMV as a model [12,32]. To the best of our knowledge, pDCs have been shown able to recognize, and respond to, all of the herpesviruses tested (Table 1). These observations suggest that pDCs may be amongst the earliest sentinels for detection of, and defense against, herpesvirus infections. The present work aims at reviewing the current knowledge regarding the role of pDCs in the orchestration of the immune responses against herpesviruses, not only focusing on their beneficial role for the host but also raising the question of their possible implications in immunopathology under defined conditions, leading to the discussion of whether and how pDC responses to herpesviruses could be used in antiviral immunotherapy or vaccination.

## 2. pDCs Contribute to IFN-I Production and Viral Control during Herpesvirus Infections

### 2.1. pDCs produce high levels of all IFN-I in vitro in response to herpesviruses

In humans, pDCs were demonstrated to be the major source of IFN-I production within total peripheral blood mononuclear cells (PBMCs) upon in vitro stimulation with HSV-1 [2]. Indeed, on a per cell basis, purified pDCs produced up to a thousand fold more IFN-I than cDCs or monocytes. Moreover, depletion of pDCs led to over a hundred fold decrease in PBMC production of IFN-I. Resting human pDCs do not contain mRNA for IFN-I. Upon HSV-1 activation, human pDCs rapidly express mRNA of all the IFN-I family members, whereas cDCs do not [38]. Under these conditions, IFN-I mRNA constitute up to 60% of the total pool of pDC neo-synthesized mRNA, attesting to the tremendous energy allocation that pDCs dedicate to the production of these antiviral cytokines upon proper stimulation. Human pDCs also produce high levels of IFN-I in vitro in response to other herpesviruses including HCMV [5–8] and EBV [11]. In mice, pDCs were likewise demonstrated to be the major source of IFN-I production within total splenic leukocytes upon in vitro stimulation with HSV-1 [35] or MCMV [39]. Among all the cells producing IFN-I in response to herpesviruses, pDCs are characterized by the fact that their IFN-I production does not require endogenous virus replication [2,40]. Moreover, pDCs are not productively infected by MCMV [13,41] or HCMV [6,7]. However, the resistance of pDCs to herpesvirus infection is not absolute. It depends both on the virus and on the source of pDCs. HHV-6 or HHV-7 productively infect blood pDCs [9,10]. Tonsil pDCs have been recently reported susceptible to HCMV infection in contrast to blood pDCs [7].

In summary, pDCs are the main IFN-I producers among total circulating leukocytes stimulated in vitro with herpesviruses. In most instances, pDC IFN-I production does not require endogenous viral replication. Indeed, human blood and mouse spleen pDCs appear resistant to productive viral infection by several herpesviruses.

### 2.2. pDCs are the major producers of IFN-I in vivo in mice infected with MCMV or HSV-2

**Figure 1.**
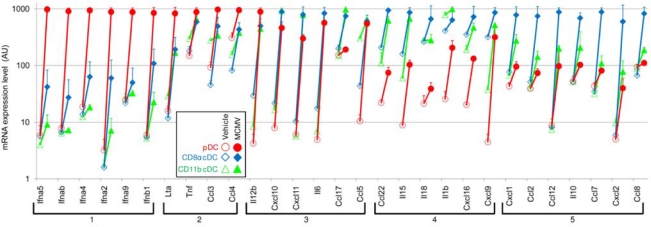
Cytokine and chemokine mRNA expression by pDCs and cDCs isolated from MCMV-infected animals at 36 hours post-challenge. pDCs (red circles), CD8α cDCs (blue diamonds) and CD11b cDCs (green triangles) were purified from C57BL/6 mice injected with vehicle (open symbols) or infected with MCMV for 36 h (closed symbols). Pangenomic microarray experiments were performed with the total mRNA isolated from these cell populations [43]. The expression of mRNA (Y-axis, in arbitrary units in log10 scale) encoding various innate cytokines and chemokines (X-axis) in the three DC subsets studied is represented. Genes were classified in 5 groups accordingly to their pattern of expression across the 6 types of biological samples examined, using the GeneCluster software. Group 1 corresponds to the IFN-I genes which are undetectable or expressed only at very low level under steady state conditions, but which are induced to extremely high levels specifically in pDCs after infection. Group 2 corresponds to genes that are induced in all DC subsets but to a higher level in pDCs. Group 3 correspond to genes that are strongly induced to a comparable extent in all three DC subsets. Group 4 corresponds to genes induced to higher levels in cDCs as compared to pDCs. Group 5 corresponds to genes induced to higher levels specifically in CD8α cDCs.

Using MCMV as a model, we were the first to report that pDCs are the major producers of IFN-I in vivo during a viral infection [12,32]. pDCs isolated from the spleen of d1.5 MCMV-infected mice produced high levels of IFN-I ex vivo without any restimulation, while the cytokines could not be detected in the supernatant from cDCs isolated from the same animals [13]. A dramatic decrease in ex vivo IFN-I production was observed in splenocytes depleted of pDCs. Moreover, systemic IFN-I production was dramatically reduced in infected animals upon anti-GR1 [12,32] or anti-BST2 [39] antibody-mediated pDC depletion. These results thus demonstrated that pDCs are the major producers of systemic IFN-I in vivo during MCMV infection, even though CD11b^+^ cDCs that are infected by MCMV in vitro at a high multiplicity of infection can also produce significant levels of IFN-I [42]. This conclusion was further confirmed by several other complementary approaches. First, pDCs isolated from the spleen of MCMV-infected mice expressed much higher amounts of the mRNA for all the different IFN-α and IFN-β subtypes tested as compared to other splenocytes [43] (Figure 1). Second, the vast majority of IFN-I expressing cells in vivo in the spleen of infected mice were demonstrated to be pDCs, using intracellular staining with specific antibodies on cell suspensions or tissue sections at 30–36 hours post-infection [43] or YFP-IFNb reporter knockin mice at 12–24 hours post-infection [44]. We showed that splenic pDCs were the major producers of IFN-I in three mouse strains: 129S2, BALB/c and C57BL/6 mice. In all these instances, IFN-I production by splenic pDCs took place relatively rapidly and was very transient since it was easily detectable around 36 hours but not at 24 hours or 44 hours post-infection [43]. Third, a deficient IFN-I production upon MCMV infection was observed in a knockin mouse strain (Ik^L/L^ mice) which harbors a selective block in pDC differentiation at an immature stage in the bone marrow consecutive to a hypomorphic mutation of the IKAROS transcription factor [45].

The intravenous infection of mice with another herpesvirus, HSV-2, also leads to a systemic IFN-I production that is abrogated upon anti-BST2 antibody-mediated pDC depletion [3]. Thus, no doubt remains that splenic pDCs are the major source of IFN-I during systemic infections of mice with several herpesviruses in vivo. Interestingly, an intra-vaginal challenge with HSV-2 also leads to a pDC-dependent IFN-I production, but only locally as the cytokines can be detected in the vaginal wash but not in the blood, consistent with the lack of dissemination of the infection in immunocompetent animals [46].

Taken together these data highlight the primordial role of pDCs as major IFN-I producers in response to systemic or local herpesvirus infections in vivo. However, this phenomenon cannot be generalized to all viruses as pDCs are not the major producers of IFN-I with certain viruses such as lymphocytic choriomeningitis virus (LCMV) [12] or upon local infections of the airways such as with intranasal Newcastle disease virus (NDV) [47] or influenza [48,49] challenges. During reovirus infections, cDCs contribute as well as pDCs to IFN-I production locally in the intestine [50]. It is noteworthy that we could not detect any IFN-I production by pDCs from the blood, lymph nodes, liver or lung of MCMV-infected mice, despite high levels of viral replication in some of these organs [43]. This is consistent with a similar observation reported by the group of Shizuo Akira in the intravenous NDV infection model [47]. This suggests that splenic pDCs are especially prone to high level IFN-I production upon systemic acute viral infections as opposed to pDCs located in some other organs such as the liver or the lung. The mechanisms for this differential responsiveness to viral stimuli of pDCs from distinct tissues are not clear, although a role of the local cytokine milieu has been proposed [51].

### 2.3. pDC-derived IFN-I contribute to the control of MCMV or HSV-2 replication in vivo, but this defense mechanism may be redundant in mouse strains with other efficient innate antiviral immune effectors

A crucial function of IFN-I is their ability to inhibit viral replication and dissemination [16]. Indeed, pDC depletion leads to enhanced local HSV-2 replication in mice infected intra-vaginally [46] and in the spleen or liver in animals infected intravenously [3], correlating with the major role of pDCs for local versus systemic IFN-I production in these experimental settings. Thus, pDC-derived IFN-I directly contributes to the control of HSV-2 replication in vivo in mice upon systemic or local infections. However, the picture appears much more complex for MCMV infection. In 129Sv mice, pDC-derived IFN-I likely contribute to MCMV control as anti-GR1 [12] or anti-BST2 (data not shown) antibody-mediated pDC depletion leads to a higher splenic viral load. In C57BL/6 mice, different approaches used to selectively affect pDC functions have led to different results. In vivo anti-GR1 [12] or anti-BST2 [39] antibody-mediated pDC depletion has no significant impact on viral replication in the spleen. Because this treatment is less efficient than in 129 mice (data not shown), it is not possible to rule out that residual pDCs still contribute sufficient IFN-I production to allow maximal antiviral effects. This interpretation could be consistent with the observation that complete abrogation of pDC IFN-I production in C57BL/6 mice leads to a significant increase in MCMV replication or in viral-induced mortality, as reported in the Ik^L/L^ mice which lack mature pDCs [45] or in interferon-regulatory factor 7 (IRF7)^−/−^ mice which have lost pDC ability to produce IFN-I [52]. However, because pDCs are not the only cell type affected in these two mutant mouse models, the interpretation of their phenotype is difficult. An alternative explanation for the differential impact of pDC depletion on MCMV replication in 129Sv versus C57BL/6 mice could be the specific capacity of the latter to mount other innate immune responses redundant for this function. Indeed, contrary to 129Sv mice, C57BL/6 mice have efficient anti-MCMV natural killer (NK) cell responses which are able to recognize and kill infected cells in vivo very rapidly after infection at the same time as pDCs are activated for IFN-I production [53]. This antiviral function of NK cells depends on their activation by IFN-I [22]. Thus, in C57BL/6 mice but not in 129Sv animals, low levels of residual IFN-I may be sufficient to promote efficient viral control by NK cells, even in the absence of strong direct antiviral effects of IFN-I, and irrespective of the cellular source of these cytokines.

In humans, several case reports have been published describing patients suffering from severe herpesvirus infections associated with decreased numbers of circulating pDCs as compared to healthy controls and with an impaired IFN-I production ability as assessed by in vitro restimulation of their PBMCs with the virus [54–56]. EBV infection of humanized NOD-SCID mice was reported to lead to a decrease in the numbers of circulating pDCs and in the ability of PBMCs to produce IFN-I upon in vitro restimulation [11]. This raises the question of whether the pDC deficiency reported in certain patients with severe herpesvirus infections could be a consequence rather than a cause of the disease. In any case, interestingly, reconstitution of NOD-SCID mice with pDC-enriched human PBMCs delayed their mortality upon consecutive infection with EBV [11]. It has been recently shown that chronic infection of mice with LCMV causes a long-lasting impairment of pDC ability to produce IFN-I upon activation with viral type stimuli and enhances sensitivity to a secondary unrelated infection with MCMV [57]. Thus, it has been proposed that enhanced sensitivity to opportunistic viruses in patients with human immunodeficiency type 1 (HIV-1) infection could in part result from decreased pDC responsiveness [57,58]. A recent study indeed shows that while IFN-I can be detected in the spleens of patients with chronic HIV-1 infection, pDCs appear to make only a very low contribution to this function [59] In this regard, it is striking that herpesviruses are amongst the most common opportunistic agents causing life-threatening disease in HIV-1-infected infants [60] or in AIDS patients [61,62] who have a dramatic reduction in pDC numbers and an impairment of pDC functions [63] in addition to the deregulation of many other immune functions. Reactivation of latent infections with herpesviruses, in particular HCMV, is also a major cause of morbidity and mortality in bone marrow or solid organ transplantation recipients [64], who undergo immunosuppressive treatments known to profoundly affect pDC numbers or functions [65–67].

In summary, manipulation of pDC numbers or functions in mice and epidemiological analyses in humans strongly suggest that high level production of IFN-I by pDCs contributes to the control of infections by herpesviruses. However, its importance could vary significantly between individuals depending on their capacity to concomitantly mount other immune responses redundant for this function. There is currently no experimental system available to the scientific community that allows the disruption of pDC functions in vivo in an entirely specific and completely efficient way. The genetic engineering of mouse models designed to fill this need should be seen as a priority in this research field, because this will be the only rigorous way to evaluate the role of pDCs in vivo in the defense against herpesviruses and more generally in the orchestration of the immune responses to viral infections. The recent demonstration that the conditional knockout of the transcription factor TCF4 (also referred to as E2-2) in adult mice leads to a complete and selective loss of pDCs constitutes a significant advance towards this goal [68].

## 3. pDC Innate Antiviral Functions Are Not Restricted to IFN-I Production but Globally Contribute to the Orchestration of Inflammation and to the Activation of Other Innate Cells

### 3.1. pDCs produce multiple cytokines and chemokines in response to viruses

Mouse splenic pDCs isolated around 1.5 days after MCMV infection of different mouse strains do not only secrete high levels of IFN-I but also produce significant amounts of IL-12p70, TNF-α, and CCL3, and induce mRNA expression for a number of other cytokines and chemokines including IL-1β, IL-6 and LTα [12,13,43] (Figure 1). Conversely, however, other cytokines that can be concomitantly detected in the spleens of infected animals seem to be mostly expressed in other specific innate immune cell types, for example IFN-γ in NK cells [13] or IL-15 in cDCs (Figure 1). Like their murine counterparts, human pDCs activated with HCMV or HSV-1 are also induced to produce and secrete many cytokines and chemokines including TNF-α, IL-6, CXCL10 and CCL3 [8,69]. However, unlike their murine counterparts, human pDCs do not express IL-12 or only at extremely low levels [31,38]. pDCs can also produce anti-inflammatory cytokines such as IL-10 in response to EBV stimulation [11].

### 3.2. pDC-derived cytokines and chemokines contribute to the activation of other innate immune effectors

The soluble factors produced by pDCs can act in a paracrine manner to recruit other cell types to the site of infection and induce them to produce a second wave of cytokines or chemokines. During MCMV infection, by producing IFN-I and CCL3, pDCs likely contribute to the induction of such a cytokine/chemokine cascade which has been demonstrated to be critical for NK cell recruitment to the sites of viral infection allowing the local delivery of their immunoregulatory and antiviral effector functions [70]. In addition, pDC-derived IL-12 directly induces IFN-γ production by NK cells [13,22] while pDC-derived IFN-I induces NK cells to proliferate [22] and to acquire cytolytic granules containing perforin and granzyme B, at least in part indirectly by instructing other cell types, most likely cDCs, to produce IL-15 and transpresent it to NK cells in association with the IL-15Rα chain [21,22,71,72]. Upon stimulation with HSV-1, pDCs have been reported to produce IL-18 and thus to most efficiently contribute to induce NK cell IFN-γ production [73]. Human pDCs stimulated with HCMV induce NK cells migration and IFN-γ secretion, but not cytotoxicity [5] which could be consistent with the poor expression of IL-15 by pDCs (Figure 1). More generally, stimulation of pDCs with herpesviruses [69] as well as with other types of viruses including hepatitis C virus [74] or influenza virus [75] induces them to produce a variety of cytokines/chemokines which recruit and activate other innate immune cell types including cDCs, monocytes and NK cells. Finally, pDCs do not contribute to the activation of other innate immune effectors solely by the production of soluble factors but also through cell-cell contacts as illustrated by the demonstration that the ligand for the glucocorticoid-induced tumor necrosis factor receptor-ligand (GITRL) is preferentially expressed on human pDCs upon HSV-1 stimulation and is critical to promote NK cell IFN-γ production and cytotoxicity [76].

**Scheme 1.**
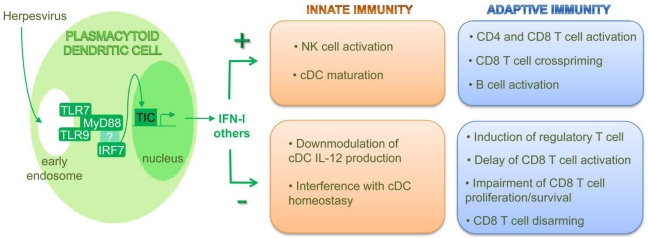
Modulation of innate and adaptive immune responses by herpesvirus-activated pDCs. pDCs are able to sense infection with herpesviruses through TLR9 and/or TLR7. The triggering of these receptors in specialized endosomes, where they are preassembled with the MyD88 adapter molecule and the IRF7 transcription factor in multimolecular complexes, leads to the phosphorylation of IRF7 and its translocation to the nucleus where it associates with other partners to constitute a transcription initiation complex (TIC) able to induce the expression of IFN-I and other target genes. Largely due to their production of innate cytokines or chemokines, pDCs can exert a variety of stimulatory or inhibitory functions on other innate or adaptive immune cell types. The global effect of pDC responses on the overall immune response and on the promotion of health versus disease depends on the combination and levels of the cytokines that they produce as discussed in Scheme 2.

In summary, although pDCs were initially characterized as professional IFN-I-producing cells upon in vitro stimulation with viruses or during viral infections in vivo, they also bear a significant contribution to the production of many other soluble factors under the same experimental settings. Thus, beyond their direct contribution to antiviral innate activities through IFN-I production, pDCs are very likely to play a critical role in the initiation and global orchestration of the recruitment and activation of other innate immune cells (Scheme 1).

## 4. pDCs Are Equipped with a Unique Molecular Machinery for Herpesvirus Recognition and Downstream High Level IFN-I Production

### 4.1. pDC activation by herpesviruses primarily relies on TLR9 recognition of unmethylated viral genomic CpG DNA sequences but RNA sensing through TLR7 can also contribute

Before pDCs were discovered, IFN-I production during viral infections was believed to mainly occur in infected cells through the activation of intra-cytoplasmic sensors for viral RNA or DNA molecules such as the RNA-dependent protein kinase (PKR) or the more recently discovered RNA helicases RIG-I and MDA-5 [77]. However, pDCs are now known to be the major producers of IFN-I upon stimulation with herpesviruses as well as some other types of viruses in vitro, and also during in vivo infections with these same viruses [3,12,43,46,78], while pDCs are most often not productively infected [4,6,13,41,79]. This apparent paradox was solved by the demonstration that pDCs express a unique set of endosomal receptors and associated signaling components allowing the detection of RNA or DNA sequences derived from engulfed viruses or infected cells. Indeed, pDCs express Toll-like receptors (TLR) 7 and 9 [80] which are localized in a specific endosomal compartment where they can respectively recognize single stranded RNA versus unmethylated CpG DNA sequences and consecutively initiate a signaling cascade ultimately resulting in high level IFN-I production. RNA or DNA molecules are not present in endosomes under steady state conditions in healthy individuals [81]. Thus, pDCs have the unique ability to detect viral infections without the requirement of endogenous viral replication, because they must be able to take up oligonucleotides from viruses or infected cells [82] into specialized endosomes for TLR7 or 9 triggering. The membrane receptors that must confer pDCs the ability to selectively recognize and engulf viral particles or apoptotic debris from infected cells remain to be identified.

MyD88 is a signaling adapter molecule used by all TLRs except TLR3 as well as by the receptors for IL-1 and IL-18. The study of MyD88^−/−^ mice has allowed to demonstrate a crucial role of this molecule for pDC IFN-I production in response to in vitro stimulations by virus type stimuli as well as in vivo during viral infections [80]. TLR9 appears to be the only MyD88-associated receptor necessary for IFN-I production during HSV-2 infection [83], which is consistent with the known presence of unmethylated CpG DNA sequences in the genome of herpesviruses. In contrast, during HSV-1 or MCMV infections, IFN-I production shows only a partial dependence on TLR9 [39,40,84,85]. This could be in part explained by the fact that other cells than pDCs contribute to IFN-I production, in a manner that is independent of MyD88 [39,84,86] but which may rely on intracellular detection of viral replication by cytoplasmic sensors for viral DNA [87] or RNA (as documented for EBV [88]). However, we have also recently shown a partial redundancy between TLR9 and TLR7 in the MyD88-dependent pDC activation for IFN-I production during MCMV infection in vivo [89]. Indeed, in TLR9^−/−^ mice, TLR7 can promote sufficient residual pDC IFN-I production to allow a better control of viral infection and an enhanced resistance to viral-induced lethality as compared to MyD88^−/−^ or TLR7^−/−^TLR9^−/−^ animals. The ability of pDCs to respond to a DNA virus through TLR7 is a strong argument in favor of their ability to engulf apoptotic bodies from dying infected cells for delivery of viral or host mRNA to TLR7-containing endosomes. Of note, TLR7 has recently been reported to be responsible for the activation of IFN-I production by cDCs upon infections with phagosomal bacteria [90]. Thus, TLR7 functions are clearly not restricted to the recognition of, and responses to, RNA viruses, but also extend to defense against herpesviruses and phagosomal bacteria. Interestingly, a cross-regulation between TLR9 and TLR7 has recently been demonstrated in mouse cDCs, whereby TLR9 outcompetes TLR7 for association with the scaffolding molecule UNC93B1 which is required for the trafficking of all endosomal TLRs from the endoplasmic reticulum to the endosomes. Thus, in wild-type animals, TLR responses in cDCs are biased towards DNA- but against RNA-sensing [91]. It will be interesting to determine whether this mechanism is also at play in pDCs, which have a particularly strong expression of TLR7 and an exquisite sensitivity to its specific ligands.

### 4.2. pDC unique ability to rapidly produce high level IFN-I results from constitutive expression of IRF7 together with TLR7/9 and MyD88 in endosomal multimolecular complexes

The unique ability of pDCs to produce very high levels of all subtypes of IFN-I in response to herpesvirus infections cannot be solely explained by their expression of TLR9 and by their ability to engulf viral particles or infected cells. Indeed, these two properties are shared with cDCs, especially the CD8α subset in the mouse, which still does not produce significant levels of IFN-I under the same experimental conditions. Indeed, additional properties have been demonstrated to contribute to the exquisite ability of pDCs to produce high levels of IFN-I upon viral stimulations (Scheme 1).

IRF7 is the master transcription factor controling IFN-I production in response to viral type stimuli, as IRF7^−/−^ mice show dramatic decreases in IFN-I production and enhanced mortality in response to challenges with different viruses, including MCMV [52] or HSV-1 [92]. Human and mouse pDCs constitutively express very high levels of IRF7 [80]. In contrast, in cDCs and other cell types, IRF7 expression is induced only secondarily to a first, IRF3-dependent, wave of IFN-β/α4 production [93] but is critical to drive the consecutive production of the other IFN-α suptypes in a positive feedback loop. Loss of IRF7 expression abolishes pDC responses to MCMV [52], HSV-1 and all the other viral stimuli that have been tested [92]. Thus, high constitutive expression of IRF7 strongly contributes to the unique ability of pDCs to produce high level of all IFN-I subtypes very rapidly upon viral stimulation. In addition, as compared to cDCs, pDCs are characterized by a long retention time of CpG desoxyoligonucleotides in dedicated, transferrin receptor^+^, endosomes where preformed multimolecular complexes exist which bridge together TLR7/9 and their downstream signaling machinery including MyD88 and IRF7 [94,95].

### 4.3. Autocrine IFN-I activity promotes high level production of these cytokines by pDCs and contributes to protect them from productive viral infection

The lack or low level of replication of certain herpesviruses and other viruses in pDCs may seem surprising considering the ability of many of these viruses to productively infect cDCs. The mechanisms that protect pDCs from productive viral infection are not entirely understood. However, autocrine IFN-I responses certainly play an important role as we initially reported by showing over a ten-fold increased proportion of infected pDCs in IFNAR^−/−^ versus WT mice challenged with an EGFP-expressing MCMV [13]. In these experimental settings, the ability to respond to IFN-I did not only protect pDCs from viral infection but also further enhanced their ability to produce IFN-I by about four-fold [12]. However, a significant production of IFN-I by pDCs still occurred in IFNAR^−/−^ animals. In mice infected with vesicular stomatitis virus (VSV), pDCs have been demonstrated to produce IFN-I in a feedback loop independent manner [96]. The impact of pDC autocrine IFN-I responses has also been studied recently in the context of NDV infection. A crucial role of this positive feedback loop was shown both for sustained IFN-I production by pDCs and for protecting them from productive viral infection [97]. Indeed, in IFNAR^−/−^ pDCs, IFN-I production is poorly sustained and entirely depends on viral replication in the pDCs and the detection of cytoplasmic viral RNA through mitochondrial antiviral signaling protein (MAV)-dependent pathways. Autocrine IFN-I activity has also been shown to protect pDCs from infection by mouse hepatitis virus [79]. Human pDCs are protected from HCMV infection due both to autocrine IFN-I effects and to other yet unidentified mechanisms [6].

The selection during evolution of a cell type specialized for IFN-I production in response to viral infection may seem paradoxical with the fact that, when productively infected, any cell type is theoretically capable of producing high levels of these cytokines, including cDCs [98]. However, the major differences between pDCs and other cell types in the molecular mechanisms in place for the sensing of the viral infections and for the downstream induction of IFN-I production are revealing. Indeed, in most instances, pDC IFN-I production does not require endogenous viral replication in the cytoplasm but only detection in endosomes of engulfed oligonucleotides derived from other infected cells or viral particles. In fact, pDCs appear resistant to productive viral infection by several herpesviruses. In addition, IFN-I production by pDCs is not strongly dependent on a positive feedback loop, because pDCs constitutively express IRF7 at very high levels. In contrast, IFN-I production by most other cell types requires detection of viral RNA or DNA in the cytoplasm, and hence endogenous productive viral replication. Under these conditions, viruses have evolved a variety of strategies to inhibit both IFN-I production and IFN-I responses in infected cells very early on after initiation of their replication [87,99–101]. Therefore, in infected cells, viruses do not only interfere with the intensity of the first wave of IFN-β/α4 but also greatly compromise the amplification loop for secondary induction of the other subtypes of IFN-α. It should be noted that HCMV has recently been reported to suppress IFN-I secretion by pDCs through its interleukin 10 homolog [102]. Nevertheless, pDCs may have a non redundant role in the rapid systemic production of IFN-I and the consecutive induction of a global innate antiviral state in the host under defined conditions of infection with herpesviruses, due to their general ability to escape viral interference with these functions.

## 5. Multiple Mechanisms Dampen pDC Responses to Viral Stimuli

Systemic innate cytokine responses can play protective antiviral functions but can also lead to severe immunopathology when too high. Thus, the benefits of activation of pDC antiviral defenses must be balanced against the risks of immunopathology. In this respect, it is likely that mechanisms are in place to finely tune the activation of pDCs depending of the nature and intensity of the environmental threats that they can perceive. The existence of such mechanisms is supported by the observation that certain autoimmune diseases including systemic lupus erythematosus (SLE) or psoriasis have been found to be associated with unbridled IFN-I production by pDCs [103–105].

### 5.1. pDCs express several endocytic receptors which signal through a specific ITAM-dependent pathway and inhibit TLR-induced cytokine production

Several studies have recently demonstrated the existence of an ITAM-dependent signaling pathway, similar to that downstream of the B cell receptor, which is triggered on human pDCs by engagement of FcεRIγ-associated membrane receptors of the C-type lectin (CLEC4C/BDCA2) or immunoglobulin (LILRA4/ILT7) super-families, and which dampens pDC cytokine responses to TLR or viral stimulations in vitro [106–108]. This regulatory pathway must be evolutionarily conserved, because some of its most specific components including BLNK and CARD11 are selectively expressed in mouse pDCs as well [28]. Indeed, the mouse SIGLECH C-type lectin receptor which associates with the ITAM-bearing adaptor DAP12 has also been shown to inhibit pDC cytokine responses to TLR stimulations [109,110]. DAP12 functions dampen pDC cytokine production [110], including in vivo in a cell autonomous way during CpG challenge or MCMV infection [111]. Thus, it is likely that the mouse SIGLECH receptor modulates pDC function through the triggering of the same B cell receptor-like intracellular signaling pathway as the human CLEC4C receptor, although these receptors are not orthologous. The natural ligands of human CLEC4C and mouse SIGLECH still remain to be identified. However, BST2 has been very recently identified in humans as a ligand for LILRA4. BST2 is a membrane molecule induced on many cell types by IFN-I [112]. Thus, human pDCs are uniquely equipped to sense the paracrine response to their production of IFN-I, allowing for a negative feedback loop to prevent excessive production of the cytokines that could cause deleterious effects to the host. Future studies will likely show that this is also the case in the mouse.

### 5.2. Other intrinsic inhibitory mechanisms contribute to dampen pDC activation

Another negative feedback effect of IFN-I has been described in pDCs that is shared with cDCs. It relies on the IFN-I-dependent induction on DCs of the TAM receptor tyrosine kinases Axl, Tyro3, or Mer. These molecules associate with the α-chain of the IFN-I receptor and activate the suppressors of cytokine signaling-1 (SOCS-1) and SOCS-3 upon engagement by their ligands Gas6 or ProS which can themselves be produced by DCs [113]. Other cell-intrinsic mechanisms exist that also contribute to dampen pDC responses to TLR triggering, such as the engagement of the immunoreceptor tyrosine-based inhibition motif (ITIM)-bearing membrane receptor DCIR in human pDCs [114]. Interestingly, engagement of DCIR on pDCs increases their capacity to present antigen to T lymphocytes. Targeted delivery of vaccine antigens to mouse pDCs via coupling to an anti-SIGLECH antibody also promotes the induction of adaptive immune responses [115]. Thus, the mechanisms that terminate the production of innate cytokines by pDCs during responses to viral type stimuli may not only be in place to prevent the development of a cytokine shock but also to switch pDC functions towards direct activation of adaptive immunity once their initial role in the orchestration of innate immune defenses has been fulfilled.

### 5.3. The control of viral replication by IFN-I and other innate immune mechanisms decreases TLR ligand availability and contributes to dampen pDC activation

In vivo during MCMV infection, pDC activation for innate cytokine production increases with the doses of viral inoculum used for the infection of a given mouse strain [116]. pDC cytokine responses to MCMV infection also increase dramatically in mice unable of efficient innate control of viral replication early after challenge, as a consequence of a selective impairment in antiviral NK cell activity, due either to antibody-dependent depletion of these cells or to the absence of the gene encoding the NK cell activation receptor specific for infected cells [116]. Reciprocally, early treatment of infected mice with an antiviral drug leads to a dramatic decrease of pDC cytokine production. Therefore, although innate cytokine production during MCMV infection is independent of endogenous viral replication in pDCs, it is tuned accordingly to the global level of viral replication in the host. Thus, rather than being the primary mechanism of defense against viral infections, high systemic production of IFN-I and other innate cytokines by spleen pDCs may represent a fail-safe mechanism. It may be turned on to maximal levels, at the risk of immunopathology, only in the case of high systemic viral replication associated to high levels of circulating ligands for TLR7 or TLR9, when other innate immune mechanisms have failed to efficiently control the virus. This hypothesis is supported by studies of airway infections of mice with viruses that do not belong to herpesviridae. For example, intranasal challenge with NDV leads to pDC production of IFN-I (in the spleen, specifically) only when the first line of defense made by alveolar macrophages is experimentally disrupted and the infection has spread systemically [47].

In summary, pDCs are equipped with a unique set of sensors and associated signaling pathways to rapidly sense a variety of viruses, in particular herpesviruses, and to respond by high level production of IFN-I and other innate cytokines or chemokines, while protecting themselves from viral infection. In this respect, pDCs appear to play an important, non redundant role in vivo in innate immune defense against herpesviruses and other viruses such as mouse hepatitis virus [78]. However, pDC activation during viral infections appears to be tightly controlled in intensity, space, and time, by a variety of mechanisms, which may be in place to tune the risk of cytokine shock to the level of threat posed to a specific host by a given viral infection.

## 6. pDCs Constitute a Link between Innate and Adaptive Immunity

pDCs are not only professional producers of IFN-I and key players in innate immune defenses against viral infections. They have also demonstrated ability to contribute to the induction and regulation of adaptive immunity. Depending on the maturation signals they receive as well as on the nature and concentration of the antigen, pDCs can have contrasting effects on adaptive immunity (Scheme 1). They can promote anti-viral cellular adaptive immunity either directly through cross-presentation of exogenous antigens to CD8 T cells or indirectly through the promotion of cDC maturation. On the contrary, under different conditions, they can induce immune tolerance.

### 6.1. pDCs can contribute to the promotion of antiviral adaptive immunity

cDCs have been long known to be specialized in antigen processing and presentation to naïve T cells leading to the induction of specific adaptive cellular immune responses. By contrast, until recently, pDCs had often been reported to be poor activators of naïve T cells, owing to their purported lower efficiency to take up, process and present exogenous antigens (reviewed in [117]). However, detailed and kinetic analyses of the expression of MHC class I and II in DC subsets upon activation have shown contrasting responses of pDCs as compared to cDCs, suggesting that these cell types function in a distinct and complementary fashion for the induction and maintenance of adaptive cellular immune responses.

Rapidly after antigen capture and receipt of maturation signals, cDCs very transiently upregulate and then drastically downmodulate antigen up-take as well as MHC class II neosynthesis and ubiquitination. This allows efficient, selective, and long-term presentation of the antigens that cDCs have encountered at the same time of receipt of the maturation signals, while the sampling and processing of the antigens encountered after maturation is shut off. Conversely, pDCs maintain MHC class II synthesis and ubiquitination after antigen capture, allowing continous sampling of the antigenic environment for processing and presentation to CD4 T cells [118,119]. Under conditions of systemic immune activation as occurs during infections with blood-borne pathogens, this specialization may allow pDCs to present to CD4 T cells some microbial antigens synthesized late after the initial encounter with the infectious agent, much more efficiently than cDCs. In the case of herpesviruses which are opportunistic pathogens, one could expect that cDCs are already activated by the primary infectious agents and are then impaired in their ability to respond to the secondary infection by the herpesviruses, whereas pDCs may still be efficient.

Recently, both mouse and human pDCs have been shown able to cross-present exogenous antigens to efficiently activate naïve or memory CD8 T cells ([120,121] and reviewed in [117]). Moreover, human pDCs have been demonstrated to harbor a unique, specialized endocytic compartment containing premade stores of MHC class I which allow rapid processing of exogenous antigens for cross-presentation to CD8 T cells [122]. Thus, it is quite possible that pDCs play a major role in the direct induction and maintenance of antiviral CD8 T cell responses during viral infections. However, very few studies have directly addressed the role of pDCs for T cell activation during infections with herpesviruses.

pDCs isolated from MCMV-infected animals up-regulate CD80, CD86, CD40 as well as MHC molecules and efficiently prime naive CD8 T cells in vitro for proliferation and IFN-γ production, when pulsed with exogenous antigens [13]. However, neither the ability of pDCs to naturally process and present MCMV antigens, nor the role of pDCs in the global shaping of antiviral CD8 T cells responses, have been addressed during the course of MCMV infection. During MCMV infection, pDC IFN-I production was required to promote cDC maturation and thus likely helped the induction of antiviral adaptive cellular immune response [13]. pDCs exposed in vitro to HSV-2 induced T cell proliferation, more strongly for CD8 T lymphocytes [4]. pDCs isolated from the draining lymph nodes of HSV-1-infected mice had only a poor ability to induce IFN-γ production by HSV-specific CD4 and CD8 T cells as compared to cDCs [123,124], suggesting that pDCs did not have a major role in the direct priming of T lymphocytes during HSV-1 infection. However, in these experimental settings, pDCs promoted optimal functions of cDCs via cell-to-cell contacts involving CD2 and CD40L, which contributed to enhance HSV-1-specific CD8 T cell responses [123]. Finally, in vitro stimulation of pDCs with HCMV or influenza promoted B cell activation and their differentiation into specific antibody-secreting plasma cells, through IFN-I- and IL-6-dependent effects [8,125].

In summary, in response to viral stimulations in vitro, or in vivo upon stimulation with synthetic TLR ligands and targeted delivery of antigens through antibody coupling, pDCs have been demonstrated to have a strong ability to cross-present exogenous antigens for the activation of naïve CD8 T cells. In addition, pDCs also play a key role in linking innate and adaptive immunity through their production of immunoregulatory cytokines or chemokines which can promote the recruitment and activation of cDCs and lymphocytes. However, the importance of these functions in vivo for the control of infections with herpesviruses still remains to be established.

### 6.2. pDCs can contribute to the negative regulation of adaptive immunity

To promote health over disease, antimicrobial immunity must be tightly regulated to allow the mounting of effector responses of sufficient strength and adequate quality for the control of the invading pathogen, but to prevent the development of immunopathology as could result from exacerbated inflammation. This delicate balance can be achieved in part through negative feedback regulatory loops acting at the level of innate immune responses as discussed earlier for pDC activation. Immunoregulatory mechanisms are also in place to finely tune adaptive immune responses, and pDCs have been demonstrated to be able to contribute to this function. In response to EBV stimulation, pDCs can produce anti-inflammatory cytokines such as IL-10 [11]. HSV-1-stimulated pDCs can downmodulate CD4 T cell activation directly through the production of IFN-I and IL-10, and indirectly through the induction of regulatory CD4 T cells, suggesting that pDCs may contribute to prevent excessive, detrimental, adaptive immune responses during viral infections [126]. The co-culture of pDCs, but not cDCs, and CD4 T cells in the presence of HCMV leads to the induction of IL-10 producing T cells [127]. pDCs have also been shown able to express the enzyme indoleamine 2,3-dioxygenase 1 (IDO), which can induce T cell tolerance through tryptophan catabolism and downstream production of pro-apoptotic metabolites [128,129]. A role for pDC expression of IDO has been demonstrated in vivo in mice for the generation of an immunosuppressive environment in tumors [130]. In humans, the expression of IDO by pDCs has been proposed to contribute the immune deregulation during HIV-1 infection [131,132]. However, to the best of our knowledge, there is currently no evidence that IDO can be expressed by pDCs in the course of infections by herpesviruses and that it can play a role in setting the balance between antiviral defense and immunopathology. The addition of an IDO inhibitor in cocultures of HSV-1-stimulated pDCs and CD4 T cells did not prevent the induction of regulatory T cells [126].

To conclude, these data showed that, in response to certain herpesviruses, pDCs can secrete anti-inflammatory cytokines and induce or activate regulatory T cells. However, these pDC functions have not yet been examined in vivo during herpesvirus infections. Regulatory T cells induced by pDCs could favor the development of highly specific adaptive immune responses targeted to the invading virus, by preventing bystander activation of T cells directed against other antigens, thereby contributing to promote antiviral defenses and to prevent immunopathology [133]. Interestingly, during intra-vaginal infections of mice with HSV-2, by controlling chemokine/cytokine production and overall immune activation in secondary lymphoid organs, regulatory T cells promote the trafficking of innate and adaptive antiviral effectors out of the lymph nodes to the site of infection for local control of viral replication [134]. Thus, taken together, these data suggest that the induction of regulatory T cells by pDCs may contribute to focus innate and adaptive antiviral immune responses to the appropriate antigens and anatomical sites to promote health over disease.

## 7. pDC response to Herpesvirus Infections Is a Double-Edged Sword: It Contributes to the Control of Viral Replication but Can also Take Part in the Induction of Immunosuppression or Immunopathology

As discussed above, pDCs are potent antiviral cells which can produce high levels of a number of cytokines or chemokines and strongly stimulate both the innate and adaptive immune systems. Although pDC functions are regulated by a number of activating or inhibitory mechanisms, situations have been described where pDC responses are deleterious for the host. The role of pDCs in the development of autoimmune diseases, including SLE or psoriasis, have been discussed in detail elsewhere [135]. Recent data have also suggested a possible deleterious role of pDCs in the context of certain viral infections. This should not be a surprise considering that IFN-I have long been known to be a double-edged sword able to promote either health or disease depending on the physiopathological context (Table 2).

For the infections with HIV-1 or simian immunodeficiency virus (SIV), there has recently been a striking paradigm shift regarding the role of pDCs in the natural history of the disease. HIV-infected individuals show decreased number of pDCs and their PBMCs are impaired for IFN-I production upon in vitro restimulation. Therefore, it was first hypothesized that a lack of pDC responses contributed to the failure to control viral replication early on after primary infection [63]. However, recently, evidences have been obtained of a massive IFN-I response during HIV-1 infection, starting early on during acute infection [136] and sustained thereafter [137,138]. This chronic production of IFN-I has been proposed to contribute to CD8 T cell disarming and uninfected CD4 T cell killing [139]. The cellular source of the IFN-I produced during chronic infection is debated [59], but pDCs could contribute [140,141]. More generally, pDCs have been reported to be hyperactivated during HIV infection and to likely contribute to immune deregulation and disease by a number of mechanisms [139,141]. Strikingly, comparisons between pDC responses of non human primate models with contrasting susceptibilities to SIV-induced disease have demonstrated much higher pDC activation in the susceptible rhesus macaques as compared to the resistant African green monkeys [142] or sooty mangabeys [138], associated with a polymorphism in the Irf7 gene between rhesus macaques and sooty mangabeys [138]. In summary, recent investigations strongly suggest that pDCs play a deleterious role for the host during infections with HIV-1 or SIV. This possibility has not yet been extensively examined during herpesvirus infections, but will be worth considering under specific conditions based on the data discussed below.

### 7.1. Herpesviruses can exploit pDC functions to promote their replication

Recently, the induction of IFN-β by TLR9 triggering on HCMV-infected fibroblasts has been reported to promote their survival and to increase their production of viral particles [154]. However, this proviral effect occurred only when fibroblasts were simulated with CpG shortly after infection. In contrast, a treatment concomitant to viral challenge almost completely abrogated fibroblast infection and virus production. Thus, it is unlikely that this mechanism plays a significant proviral role to the disadvantage of the host during infections with most herpesviruses in vivo, as systemic production of IFN-I by pDCs should lead to the induction of antiviral defenses in most cells before they encounter the virus. However, the possibility remains that this mechanism could have been exploited by herpesviruses which can productively infect pDCs such as HHV-6 and HHV-7 [9,10]. Some data suggest that pDCs could participate to the local replication and to the dissemination of HHV-6 and HHV-7, as skin lesions induced by the infections with these viruses have been reported to be infiltrated by infected pDCs [10], and infected pDCs can transmit the virus to stimulated T cells [9].

### 7.2. Excessive pDC activation during viral infections can contribute to immunosuppression or lead to immunopathology

As mentioned earlier, IFN-I can promote either health or disease depending on the physiopathological context (Table 2). Therefore, high level production of IFN-I by pDCs such as occurs during certain herpesvirus infections could contribute to immunosuppression or even lead to immunopathology. Indeed, we have shown that high levels of pDC innate cytokine production during MCMV infection are associated with an ablation of cDCs and a significant delay in the induction of antiviral CD8 T cell responses for up to 48 hours [116]. This transient immunosuppression can be recapitulated by exogenous administration of IFN-I in mouse strains that mount low pDC responses to MCMV infection, pointing to a crucial role of pDC-derived IFN-I in this phenomenon [116]. This impact of pDC functions on the kinetics of induction of antiviral adaptive immunity is striking given the recent observation that better outcomes of early primary viral infection with LCMV in mice or SIV in macaques correlate with increased ratio between antigen-specific CD8 T cells and infected cells in situ very early after challenge [155]. TNF-α has been shown to contribute to the disease induced by MCMV infection, as it contributes to liver pathology independently of NK and T cell responses [156]. pDCs could also contribute to these effects since they are the major source of TNF-α early after infection in different mouse strains [43].

### 7.3. Is-there a role for herpesvirus-dependent pDC activation in the triggering of certain autoimmune diseases?

Because chronic IFN-I or TNF-α responses often occur in a variety of autoimmune diseases [157], it will be worth examining whether pDC activation by herpesvirus infections may contribute to the development of autoimmunity. As mentioned earlier, a deleterious role of pDCs has been proposed for SLE or psoriasis [103,104]. Although the etiology of these diseases is not entirely understood, it is possible that infections with certain herpesviruses could initiate the pathology in some patients. The possible implication of EBV infection in several autoimmune diseases including SLE is indeed debated [158,159].

## 8. From the Bench to the Bedside: Exploiting pDC Responses to Herpesvirus Infections for Therapeutic Purposes

### 8.1. Boosting pDC antiviral responses

**Scheme 2.**
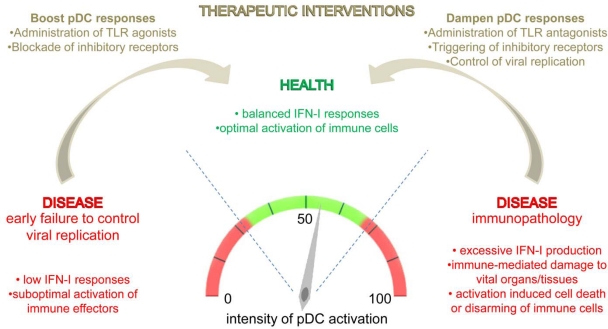
Impact of the fine tuning of pDC activation on the promotion of health versus disease. During herpesvirus infections, disease can result either from immune failure to control viral replication early or from the development of immunopathology. Weak pDC activation could contribute to disease in the former case, and excessive pDC activation in the latter case. Immunopathology can cause immune-mediated damage to vital organs and/or compromise the ability of adaptive immunity to control viral replication later. Thus, a significant but controlled pDC activation is required to promote health over disease, by allowing early control of viral replication while not causing significant immunopathology. Therapeutic protocols aimed at boosting or dampening pDC responses could thus help to reach this balance and to fight disease under defined clinical conditions, as discussed in the body of this review.

Studies in mice have demonstrated a TLR9- and MyD88-dependent protective role of local CpG administration following intravaginal infection with HSV-2 [160]. pDCs were recruited in the vaginal mucosa 24 hours after CpG treatment. Anti-BST2 antibody-mediated pDC depletion in vivo abrogated the protection. In humans with genital infection by HSV, topical application on the affected regions of a cream formulation of a TLR7 agonist, imiquimod, allows remission of the lesions [161], likely through the local recruitment and activation of pDCs for IFN-I production. Importantly, this treatment has proven efficient even in the case of recurrent lesions caused by viruses resistant to the classical antiviral drug acyclovir [161]. This was the case for an AIDS-patient whose pDCs were unable to respond to HSV-1 upon in vitro restimulation but produced high levels of IFN-I upon exposure to imiquimod [54]. Thus, boosting pDC responses on genital lesions caused by herpesviruses, through the local administration of TLR7 or 9 agonists, can lead to efficient control of the virus and cure of the disease through activation of antiviral innate immunity, in complement or alternatively to the use of classical antiviral drugs directly inhibiting the viral DNA polymerase (Scheme 2).

In mice, boosting pDC responses has also been shown to enhance resistance to systemic infection with MCMV or HSV-1. This has been achieved either through the systemic administration of the Thymosin-α1 peptide [41] or of FLT3L [162]. In both cases, enhanced protection against the viral infection resulted from increased IFN-I or IL-12 production. For the MCMV infection, this was shown to depend on the activation of the TLR9/MyD88/IRF7 pathway in pDCs and to lead to a higher NK cell activation. Since a deficiency in pDC responses has been proposed to be implicated in the susceptibility of bone marrow or solid organ transplant recipients to HCMV reactivation or primary infection [65,67], therapeutic strategies aimed at restoring normal pDC functions in this physiopathological context may bring clinical benefits (Scheme 2). However, as compared to the local activation of pDCs in skin or mucosa by topical application of TLR agonists, systemic activation of pDCs may bear increased risks of detrimental side effects. Thus, further studies will be required to determine the best strategy to harness pDCs for the promotion of efficient systemic innate antiviral defenses without triggering excessive inflammation and consecutive immunopathology.

### 8.2. Dampening pDC activation

As mentioned above, excessive pDC activation during infections with HIV-1/SIV or with MCMV can lead to an overwhelming production of IFN-I and other innate cytokines. This in turn can lead to immune deregulation, including delaying or disarming of adaptive immunity, or to the generation of a local inflammation which contributes to support viral replication and dissemination [163]. In addition, pDCs have been involved in autoimmune diseases for which certain herpesvirus infections have been suggested to be potential triggers [158,159]. Thus, the dampening of pDC responses could be a reasonable component of immunotherapeutic strategies aimed at decreasing the immunopathology in these different conditions (Scheme 2). Interestingly, in a model of genital infection of macaques with SIV, the dampening of the pDC response locally in the vagina resulted in reduced local inflammation, including a decreased CD4 T cell infiltration, and led to a significant protection of the animals against repeated high dose challenge with SIV [163]. These effects were achieved by local application of glycerol monolaurate which dampened the activation of endocervical epithelial cells for CCL20 production and therefore prevented the downstream CCR6–dependent recruitment of pDCs in the vagina. Dampening systemic pDC responses in vivo could theoretically be achieved by a number of ways, including treatments with antagonist ligands of the TLR receptors [164], or the administration of agonistic antibodies to the LILRA4 or CLEC4C receptors which are selectively expressed in pDCs and downmodulate their production of innate cytokines in response to TLR triggering [106–108]. However, it must be stressed that pDC responses should not be completely switched off, because this may lead to heightened viral replication, as recently demonstrated for inflammatory DCs in the case of a mouse model of intranasal influenza infection. Indeed, in this model, either excessive or absent inflammatory DC responses turned out to be deleterious for the host, due respectively to immunopathology versus loss of control over viral replication. In contrast, dampening of inflammatory DC responses through drug treatment allowed enhanced resistance to disease [165]. Thus, therapies aimed at dampening pDC responses to limit immunopathology in the course of viral infections should be combined with other treatments aimed at controling viral replication, such as antiviral drugs or immunotherapeutic restoration of the cytotoxic antiviral activity of NK cells or CD8 T lymphocytes.

### 8.3. Exploiting herpesviruses as potent vectors for vaccination against other intracellular pathogens

Herpesviruses are well controlled by immunocompetent hosts, since most humans are infected by HCMV, EBV, or HSV, and yet do not develop any clinical symptoms. All three of these viruses appear particularly efficient at activating pDCs. Therefore, it seems reasonable to consider that pDCs may contribute to the induction of the strong, protective, adaptive immune responses observed to occur naturally in most infected individuals. Interestingly, the ability to trigger a variety of TLRs and to activate multiple DC subsets, including pDCs, is part of the explanation that has been proposed for the high efficacy of the attenuated yellow fever virus vaccine [166]. In the case of herpesviruses, other factors are also likely to contribute to the induction of long-lasting protective adaptive immunity. In particular, this is the case of the inflation over time of the memory CD8 T cells directed against the products of immediate early genes [167]. This inflation occurs due to the natural boosts provided by expression of these antigens during partial reactivation of virus from latently infected cells over the lifetime of the infected individuals [168]. Therefore, it would be worth considering the exploitation of herpesviruses as potent vectors for vaccination against other intracellular pathogens or tumors. Indeed, this has been already done rather successfully in mouse models of vaccination against influenza or LCMV, where the target antigens had been introduced under the control of the promoter of the gene encoding the immediate early antigen-2 [169] as well as very recently in rhesus macaques for vaccination against SIV although expression of the vaccine antigens had been driven by the promoter of a structural gene which should not induce CD8 T cell memory inflation [170]. The role of pDCs in the success of these vaccination protocols would be interesting to evaluate.

## 9. Conclusion

The various studies on pDCs in the context of infections by herpesviruses show a strong involvement of these cells in the antiviral immune response. Corresponding key issues are summarized in Table 3. Both in vitro and in vivo, pDCs are the major source of IFN-I during stimulations or infections by herpesviruses. IFN-I responses have been shown to play a critical role in the control of these viral infections, in particular in the case of MCMV or HSV2. pDCs have a unique ability to rapidly produce high and systemic levels of IFN-I, without the requirement for endogenous viral replication, upon engulfment of viral products. This can be explained by the constitutive expression in pDCs of a specific set of viral sensors, including TLR7 and TLR9, and of downstream signaling molecules including MyD88 and IRF7, arranged as preassembled multimolecular complexes in special endosomes. Although the expression of some of these molecules can be shared with other immune cell subsets, this is not the case of the whole set altogether as a preassembled endosomal multimolecular complex. In addition, pDCs are also able to produce multiple other cytokines or chemokines upon activation by herpesviruses. Thus, pDCs are likely to play a non redundant role in the induction and regulation of innate immune responses to herpesviruses. It should be noted that humans with genetic deficiencies in signaling components known to be critical for pDC IFN-I production in response to viral stimulation do not appear to suffer from life threatening viral infections, except for herpes simplex encephalitis [171,172]. This suggests that constitutive lack of pDC-derived IFN-I does not compromise the ultimate control of herpesvirus infections in humans in the absence of generalized immunodeficiency and in the context of the advanced health care system of developed countries. However, very recently, the sequencing and analysis of the ten human TLRs in over 150 individuals from various ethnicity demonstrated that the endosomal TLRs have evolved under strong purifying selection, including TLR7 and TLR9 which are selectively expressed to high levels in pDCs in humans. This suggests an essential non-redundant role of the endosomal TLRs in host survival “either via protective immunity against viral infections (present or past), or because of their additional involvement in other non immunity related processes of major biological relevance, or both” [173]. In any case, the possibility to harness pDC functions in the clinic to help treat infections with herpesviruses in immunocompromised individuals is promising. Topical treatments of genital lesions due to recurrent herpesvirus infections have indeed already given encouraging results. The role of pDCs in the regulation of adaptive immune responses to herpesvirus infections is less well understood. It is clear that pDCs can promote antiviral CD8 T cell responses through cDC instruction by soluble factors as well as cell-to-cell contacts. Whether pDCs bear a significant contribution to the direct priming of antiviral T lymphocytes in vivo during viral infections, as compared to cDCs, remains an open question on which future work will likely focus in the coming years. Moreover, pDCs are also able of inducing immunosuppression or tolerance under specific experimental conditions, which has not been yet studied in the context of infections with herpesviruses in vivo.

While attention has been mainly drawn on the goods of pDC responses to herpesviruses, one should not ignore the possibility that pDCs may also contribute to disease for given herpesviruses in specific physiopathological contexts. From this point of view, it is revealing that for infections with HIV-1 or SIV, a paradigm shift recently occurred regarding the role of pDCs in the disease. In contrast to the initial hypothesis that pDC responses should be protective, the current idea is that pDCs play an important deleterious role in the development of the disease. In the mouse model of MCMV infection, pDC hyperactivation can contribute to induce a transient suppression of antiviral adaptive immunity likely due in part to deleterious effects of high systemic levels of IFN-I on cDCs or CD8 T cells. pDCs have also been shown to be involved in a variety of autoimmune diseases for which infections with certain herpesviruses may constitute possible triggering agents. Under these physiopathological settings, therapeutic strategies aimed at dampening pDC activation may yield a clinical benefit.

Significant progress has been made in the last couple of years on the deciphering of the innate functions of pDCs and their regulation during viral infections in vivo. However, further studies are required to improve our knowledge of the overall role of pDCs in the physiopathology of the infections with herpesviruses as well as other viruses, and to deepen our understanding of how the positive and negative mechanisms which regulate pDC functions integrate in time and space to promote health over disease. To this aim, the generation of novel mouse models specifically devoid of pDCs, or selectively affected in their functions, will be instrumental. In addition, the comparison of the outcome of herpesvirus infections and of the antiviral immune responses between non human primate models naturally differing in their pDC responses to TLR stimulations, such as macaques and sooty mangabeys [138], would be enlightening.

## Figures and Tables

**Table 1.** Most common parameters measured to study the impact of herpesvirus stimulation on pDC biology.

			**Impact on pDC biology**		
**Herpesviruses**		**natural host**	**IFN-I production**	**Infection**	**Maturation^$^**
Herpes Simplex virus 1	HSV-1	human	Yes* [2]	ND	ND
Herpes Simplex virus 2	HSV-2	human	Yes*^±^ [3]	No [4]	ND
Varicella-zooster virus	VZV	human	ND	ND	ND
Human cytomegalovirus	HCMV	human	Yes [5,6,7]	No (blood pDCs) [6]	Partial [8]
				Yes (tonsil pDCs) [7]	
Human Herpesvirus 6	HHV-6	human	ND	Yes [9]	ND
Human Herpesvirus 7	HHV-7	human	ND	Yes [10]	ND
Epstein-Barr virus	EBV	human	Yes [11]	ND	Yes [11]
Human Herpesvirus 8	HHV-8	human	ND	ND	ND
Murine cytomegalovirus	MCMV	mouse	Yes*^±^ [12]	No^±^ [13]	Yes^±^ [13]

* pDCs have been described to be the main IFN-I source upon herpesvirus stimulation.

± including in vivo in mice

$ maturation of pDCs following stimulation with herpesviruses in terms of CD40, CD80, CD86 and MHCII up-regulation

ND: not determined

**Table 2.** IFN-I responses to viral infections are a double edged sword.

The goods of IFN-I responses	Ref.	The bads of IFN-I responses	Ref.
Direct antiviral effects	[15]	Development of autoimmunity or immunopathology	[15,143]
Promotion of cDC maturation	[13,23]	Induction of DC apoptosis	[144]
promotion of cDC cross-presentation	[24]	Prevention of DC renewal	[145]
promotion of NK cell activation	[19,22]	General inhibition of hematopoiesis	[146,147]
Help to CD8 T lymphocytes	[17,18,20]	Anti-proliferative or pro-apoptotic effects on CD8 T cells	[15,148,149]
Help to B lymphocytes	[150,151]	Susceptibility to bacterial surinfections	[152,153]

**Table 3.** Key issues.

Major concepts and outstanding questions regarding pDC responses to herpesviruses
IFN-I are innate cytokines endowed with potent direct and indirect antiviral activities. The expression of the receptor for IFN-I is ubiquitous and allows widespread systemic effects of the cytokines. IFN-I responses are complex and can induce protective antiviral responses or immunopathology depending on the timing, level and anatomical site of their production. Herpesviruses can interfere with the induction of, or the responses to, IFN-I to escape immunity. pDCs are the main IFN-I producers in response to many viruses including all the herpesviruses tested. pDCs are able to sense herpesvirus infections through the TLR7/9 receptors in a MyD88 dependant manner. pDCs produce a large panel of cytokines/chemokines and thus must play a major role in the orchestration of early inflammation and downstream activation of innate and adaptive immune effectors. Mature pDCs can cross-present viral antigens for cognate CD8 T cell activation. Excessive pDC activation during viral infections can contribute to immunopathology. It is not known whether pDC responses to common herpesvirus infections could contribute to the development of certain autoimmune diseases in susceptible individuals. It is not known whether, and how, pDCs are required for the induction and polarization of T cell responses during herpesvirus infections in vivo. To rigorously evaluate the multiple roles of pDCs in vivo in the orchestration of antiviral immune responses, the genetic engineering of novel mouse models specifically devoid of pDCs or selectively affected in their functions will be crucial. pDCs are interesting targets for the design of novel immunotherapeutic approaches against viral infections.

## References

[1] Schleiss MR (2009). Persistent and recurring viral infections: the human herpesviruses. Curr Probl Pediatr Adolesc Health Care.

[2] Siegal FP, Kadowaki N, Shodell M, Fitzgerald-Bocarsly PA, Shah K, Ho S, Antonenko S, Liu YJ (1999). The nature of the principal type 1 interferon-producing cells in human blood. Science.

[3] Stout-Delgado HW, Yang X, Walker WE, Tesar BM, Goldstein DR (2008). Aging impairs IFN regulatory factor 7 up-regulation in plasmacytoid dendritic cells during TLR9 activation. J Immunol.

[4] Donaghy H, Bosnjak L, Harman AN, Marsden V, Tyring SK, Meng TC, Cunningham AL (2009). Role for plasmacytoid dendritic cells in the immune control of recurrent human herpes simplex virus infection. J Virol.

[5] Cederarv M, Soderberg-Naucler C, Odeberg J (2009). HCMV infection of PDCs deviates the NK cell response into cytokine-producing cells unable to perform cytotoxicity. Immunobiology.

[6] Kvale EO, Dalgaard J, Lund-Johansen F, Rollag H, Farkas L, Midtvedt K, Jahnsen FL, Brinchmann JE, Olweus J (2006). CD11c+ dendritic cells and plasmacytoid DCs are activated by human cytomegalovirus and retain efficient T cell-stimulatory capability upon infection. Blood.

[7] Schneider K, Meyer-Koenig U, Hufert FT (2008). Human cytomegalovirus impairs the function of plasmacytoid dendritic cells in lymphoid organs. PLoS One.

[8] Varani S, Cederarv M, Feld S, Tammik C, Frascaroli G, Landini MP, Soderberg-Naucler C (2007). Human cytomegalovirus differentially controls B cell and T cell responses through effects on plasmacytoid dendritic cells. J Immunol.

[9] Takemoto M, Imasawa T, Yamanishi K, Mori Y (2009). Role of dendritic cells infected with human herpesvirus 6 in virus transmission to CD4(+) T cells. Virology.

[10] de Vries HJ, Teunissen MB, Zorgdrager F, Picavet D, Cornelissen M (2007). Lichen planus remission is associated with a decrease of human herpes virus type 7 protein expression in plasmacytoid dendritic cells. Arch Dermatol Res.

[11] Lim WH, Kireta S, Russ GR, Coates PT (2007). Human plasmacytoid dendritic cells regulate immune responses to Epstein-Barr virus (EBV) infection and delay EBV-related mortality in humanized NOD-SCID mice. Blood.

[12] Dalod M, Salazar-Mather TP, Malmgaard L, Lewis C, Asselin-Paturel C, Briere F, Trinchieri G, Biron CA (2002). Interferon alpha/beta and interleukin 12 responses to viral infections: pathways regulating dendritic cell cytokine expression in vivo. J Exp Med.

[13] Dalod M, Hamilton T, Salomon R, Salazar-Mather TP, Henry SC, Hamilton JD, Biron CA (2003). Dendritic cell responses to early murine cytomegalovirus infection: subset functional specialization and differential regulation by interferon alpha/beta. J Exp Med.

[14] Isaacs A, Lindenmann J (1957). Virus interference. I. The interferon. Proc R Soc Lond B Biol Sci.

[15] Theofilopoulos AN, Baccala R, Beutler B, Kono DH (2005). Type I interferons (alpha/beta) in immunity and autoimmunity. Annu Rev Immunol.

[16] Garcia-Sastre A, Biron CA (2006). Type 1 interferons and the virus-host relationship: a lesson in detente. Science.

[17] Aichele P, Unsoeld H, Koschella M, Schweier O, Kalinke U, Vucikuja S (2006). CD8 T cells specific for lymphocytic choriomeningitis virus require type I IFN receptor for clonal expansion. J Immunol.

[18] Le Bon A, Durand V, Kamphuis E, Thompson C, Bulfone-Paus S, Rossmann C, Kalinke U, Tough DF (2006). Direct stimulation of T cells by type I IFN enhances the CD8+ T cell response during cross-priming. J Immunol.

[19] Martinez J, Huang X, Yang Y (2008). Direct action of type I IFN on NK cells is required for their activation in response to vaccinia viral infection in vivo. J Immunol.

[20] Kolumam GA, Thomas S, Thompson LJ, Sprent J, Murali-Krishna K (2005). Type I interferons act directly on CD8 T cells to allow clonal expansion and memory formation in response to viral infection. J Exp Med.

[21] Lucas M, Schachterle W, Oberle K, Aichele P, Diefenbach A (2007). Dendritic cells prime natural killer cells by trans-presenting interleukin 15. Immunity.

[22] Nguyen KB, Salazar-Mather TP, Dalod MY, Van Deusen JB, Wei XQ, Liew FY, Caligiuri MA, Durbin JE, Biron CA (2002). Coordinated and distinct roles for IFN-alpha beta, IL-12, and IL-15 regulation of NK cell responses to viral infection. J Immunol.

[23] Honda K, Sakaguchi S, Nakajima C, Watanabe A, Yanai H, Matsumoto M, Ohteki T, Kaisho T, Takaoka A, Akira S, Seya T, Taniguchi T (2003). Selective contribution of IFN-alpha/beta signaling to the maturation of dendritic cells induced by double-stranded RNA or viral infection. Proc Natl Acad Sci U S A.

[24] Le Bon A, Tough DF (2008). Type I interferon as a stimulus for cross-priming. Cytokine Growth Factor Rev.

[25] Pestka S, Krause CD, Walter MR (2004). Interferons, interferon-like cytokines, and their receptors. Immunol Rev.

[26] Blasius AL, Barchet W, Cella M, Colonna M (2007). Development and function of murine B220+CD11c+NK1.1+ cells identify them as a subset of NK cells. J Exp Med.

[27] Caminschi I, Ahmet F, Heger K, Brady J, Nutt SL, Vremec D, Pietersz S, Lahoud MH, Schofield L, Hansen DS, O’Keeffe M, Smyth MJ, Bedoui S, Davey GM, Villadangos JA, Heath WR, Shortman K (2007). Putative IKDCs are functionally and developmentally similar to natural killer cells, but not to dendritic cells. J Exp Med.

[28] Robbins SH, Walzer T, Dembele D, Thibault C, Defays A, Bessou G, Xu H, Vivier E, Sellars M, Pierre P, Sharp FR, Chan S, Kastner P, Dalod M (2008). Novel insights into the relationships between dendritic cell subsets in human and mouse revealed by genome-wide expression profiling. Genome Biol.

[29] Vosshenrich CA, Lesjean-Pottier S, Hasan M, Richard-Le Goff O, Corcuff E, Mandelboim O, Di Santo JP (2007). CD11cloB220+ interferon-producing killer dendritic cells are activated natural killer cells. J Exp Med.

[30] Segura E, Wong J, Villadangos JA (2009). Cutting edge: B220+CCR9- dendritic cells are not plasmacytoid dendritic cells but are precursors of conventional dendritic cells. J Immunol.

[31] Liu YJ (2005). IPC: professional type 1 interferon-producing cells and plasmacytoid dendritic cell precursors. Annu Rev Immunol.

[32] Asselin-Paturel C, Boonstra A, Dalod M, Durand I, Yessaad N, Dezutter-Dambuyant C, Vicari A, O’Garra A, Biron C, Briere F, Trinchieri G (2001). Mouse type I IFN-producing cells are immature APCs with plasmacytoid morphology. Nat Immunol.

[33] Cella M, Jarrossay D, Facchetti F, Alebardi O, Nakajima H, Lanzavecchia A, Colonna M (1999). Plasmacytoid monocytes migrate to inflamed lymph nodes and produce large amounts of type I interferon. Nat Med.

[34] Nakano H, Yanagita M, Gunn MD (2001). CD11c(+)B220(+)Gr-1(+) cells in mouse lymph nodes and spleen display characteristics of plasmacytoid dendritic cells. J Exp Med.

[35] Bjorck P (2001). Isolation and characterization of plasmacytoid dendritic cells from Flt3 ligand and granulocyte-macrophage colony-stimulating factor-treated mice. Blood.

[36] Chehimi J, Starr SE, Kawashima H, Miller DS, Trinchieri G, Perussia B, Bandyopadhyay S (1989). Dendritic cells and IFN-alpha-producing cells are two functionally distinct non-B, non-monocytic HLA-DR+ cell subsets in human peripheral blood. Immunology.

[37] Fitzgerald-Bocarsly P, Feldman M, Mendelsohn M, Curl S, Lopez C (1988). Human mononuclear cells which produce interferon-alpha during NK(HSV-FS) assays are HLA-DR positive cells distinct from cytolytic natural killer effectors. J Leukoc Biol.

[38] Ito T, Kanzler H, Duramad O, Cao W, Liu YJ (2006). Specialization, kinetics, and repertoire of type 1 interferon responses by human plasmacytoid predendritic cells. Blood.

[39] Krug A, French AR, Barchet W, Fischer JA, Dzionek A, Pingel JT, Orihuela MM, Akira S, Yokoyama WM, Colonna M (2004). TLR9-dependent recognition of MCMV by IPC and DC generates coordinated cytokine responses that activate antiviral NK cell function. Immunity.

[40] Rasmussen SB, Sorensen LN, Malmgaard L, Ank N, Baines JD, Chen ZJ, Paludan SR (2007). Type I interferon production during herpes simplex virus infection is controlled by cell-type-specific viral recognition through Toll-like receptor 9, the mitochondrial antiviral signaling protein pathway, and novel recognition systems. J Virol.

[41] Bozza S, Gaziano R, Bonifazi P, Zelante T, Pitzurra L, Montagnoli C, Moretti S, Castronari R, Sinibaldi P, Rasi G, Garaci E, Bistoni F, Romani L (2007). Thymosin alpha1 activates the TLR9/MyD88/IRF7-dependent murine cytomegalovirus sensing for induction of anti-viral responses in vivo. Int Immunol.

[42] Andoniou CE, van Dommelen SL, Voigt V, Andrews DM, Brizard G, Asselin-Paturel C, Delale T, Stacey KJ, Trinchieri G, Degli-Esposti MA (2005). Interaction between conventional dendritic cells and natural killer cells is integral to the activation of effective antiviral immunity. Nat Immunol.

[43] Zucchini N, Bessou G, Robbins SH, Chasson L, Raper A, Crocker PR, Dalod M (2008). Individual plasmacytoid dendritic cells are major contributors to the production of multiple innate cytokines in an organ-specific manner during viral infection. Int Immunol.

[44] Scheu S, Dresing P, Locksley RM (2008). Visualization of IFNbeta production by plasmacytoid versus conventional dendritic cells under specific stimulation conditions in vivo. Proc Natl Acad Sci U S A.

[45] Allman D, Dalod M, Asselin-Paturel C, Delale T, Robbins SH, Trinchieri G, Biron CA, Kastner P, Chan S (2006). Ikaros is required for plasmacytoid dendritic cell differentiation. Blood.

[46] Lund JM, Linehan MM, Iijima N, Iwasaki A (2006). Cutting Edge: Plasmacytoid dendritic cells provide innate immune protection against mucosal viral infection in situ. J Immunol.

[47] Kumagai Y, Takeuchi O, Kato H, Kumar H, Matsui K, Morii E, Aozasa K, Kawai T, Akira S (2007). Alveolar macrophages are the primary interferon-alpha producer in pulmonary infection with RNA viruses. Immunity.

[48] GeurtsvanKessel CH, Willart MA, van Rijt LS, Muskens F, Kool M, Baas C, Thielemans K, Bennett C, Clausen BE, Hoogsteden HC, Osterhaus AD, Rimmelzwaan GF, Lambrecht BN (2008). Clearance of influenza virus from the lung depends on migratory langerin+CD11b- but not plasmacytoid dendritic cells. J Exp Med.

[49] Wolf AI, Buehler D, Hensley SE, Cavanagh LL, Wherry EJ, Kastner P, Chan S, Weninger W (2009). Plasmacytoid dendritic cells are dispensable during primary influenza virus infection. J Immunol.

[50] Johansson C, Wetzel JD, He J, Mikacenic C, Dermody TS, Kelsall BL (2007). Type I interferons produced by hematopoietic cells protect mice against lethal infection by mammalian reovirus. J Exp Med.

[51] Contractor N, Louten J, Kim L, Biron CA, Kelsall BL (2007). Cutting edge: Peyer’s patch plasmacytoid dendritic cells (pDCs) produce low levels of type I interferons: possible role for IL-10, TGFbeta, and prostaglandin E2 in conditioning a unique mucosal pDC phenotype. J Immunol.

[52] Steinberg C, Eisenacher K, Gross O, Reindl W, Schmitz F, Ruland J, Krug A (2009). The IFN regulatory factor 7-dependent type I IFN response is not essential for early resistance against murine cytomegalovirus infection. Eur J Immunol.

[53] Scalzo AA, Yokoyama WM (2008). Cmv1 and natural killer cell responses to murine cytomegalovirus infection. Curr Top Microbiol Immunol.

[54] Abbo L, Vincek V, Dickinson G, Shrestha N, Doblecki S, Haslett PA (2007). Selective defect in plasmacyoid dendritic cell function in a patient with AIDS-associated atypical genital herpes simplex vegetans treated with imiquimod. Clin Infect Dis.

[55] Dalloul A, Oksenhendler E, Chosidow O, Ribaud P, Carcelain G, Louvet S, Massip P, Lebon P, Autran B (2004). Severe herpes virus (HSV-2) infection in two patients with myelodysplasia and undetectable NK cells and plasmacytoid dendritic cells in the blood. J Clin Virol.

[56] Kittan NA, Bergua A, Haupt S, Donhauser N, Schuster P, Korn K, Harrer T, Schmidt B (2007). Impaired plasmacytoid dendritic cell innate immune responses in patients with herpes virus-associated acute retinal necrosis. J Immunol.

[57] Zuniga EI, Liou LY, Mack L, Mendoza M, Oldstone MB (2008). Persistent virus infection inhibits type I interferon production by plasmacytoid dendritic cells to facilitate opportunistic infections. Cell Host Microbe.

[58] Baranek T, Dalod M (2008). How opportunistic agents benefit from viral infections: the plasmacytoid dendritic cell connection. Cell Host Microbe.

[59] Nascimbeni M, Perie L, Chorro L, Diocou S, Kreitmann L, Louis S, Garderet L, Fabiani B, Berger A, Schmitz J, Marie JP, Molina TJ, Pacanowski J, Viard JP, Oksenhendler E, Beq S, Abehsira-Amar O, Cheynier R, Hosmalin A (2009). Plasmacytoid dendritic cells accumulate in spleens from chronically HIV-infected patients but barely participate in interferon-alpha expression. Blood.

[60] Slyker JA, Lohman-Payne BL, John-Stewart GC, Maleche-Obimbo E, Emery S, Richardson B, Dong T, Iversen AK, Mbori-Ngacha D, Overbaugh J, Emery VC, Rowland-Jones SL (2009). Acute cytomegalovirus infection in Kenyan HIV-infected infants. AIDS.

[61] Gallant JE, Moore RD, Richman DD, Keruly J, Chaisson RE (1992). Incidence and natural history of cytomegalovirus disease in patients with advanced human immunodeficiency virus disease treated with zidovudine. The Zidovudine Epidemiology Study Group. J Infect Dis.

[62] Van de Perre P, Segondy M, Foulongne V, Ouedraogo A, Konate I, Huraux JM, Mayaud P, Nagot N (2008). Herpes simplex virus and HIV-1: deciphering viral synergy. Lancet Infect Dis.

[63] Hosmalin A, Lebon P (2006). Type I interferon production in HIV-infected patients. J Leukoc Biol.

[64] Britt W (2008). Manifestations of human cytomegalovirus infection: proposed mechanisms of acute and chronic disease. Curr Top Microbiol Immunol.

[65] Giraud S, Dhedin N, Gary-Gouy H, Lebon P, Vernant JP, Dalloul A (2005). Plasmacytoid dendritic cell reconstitution following bone marrow transplantation: subnormal recovery and functional deficit of IFN-alpha/beta production in response to herpes simplex virus. J Interferon Cytokine Res.

[66] Abe M, Thomson AW (2006). Dexamethasone preferentially suppresses plasmacytoid dendritic cell differentiation and enhances their apoptotic death. Clin Immunol.

[67] Boor PP, Metselaar HJ, Mancham S, Tilanus HW, Kusters JG, Kwekkeboom J (2006). Prednisolone suppresses the function and promotes apoptosis of plasmacytoid dendritic cells. Am J Transplant.

[68] Cisse B, Caton ML, Lehner M, Maeda T, Scheu S, Locksley R, Holmberg D, Zweier C, den Hollander NS, Kant SG, Holter W, Rauch A, Zhuang Y, Reizis B (2008). Transcription factor E2-2 is an essential and specific regulator of plasmacytoid dendritic cell development. Cell.

[69] Megjugorac NJ, Young HA, Amrute SB, Olshalsky SL, Fitzgerald-Bocarsly P (2004). Virally stimulated plasmacytoid dendritic cells produce chemokines and induce migration of T and NK cells. J Leukoc Biol.

[70] Salazar-Mather TP, Hokeness KL (2006). Cytokine and chemokine networks: pathways to antiviral defense. Curr Top Microbiol Immunol.

[71] Fehniger TA, Cai SF, Cao X, Bredemeyer AJ, Presti RM, French AR, Ley TJ (2007). Acquisition of murine NK cell cytotoxicity requires the translation of a pre-existing pool of granzyme B and perforin mRNAs. Immunity.

[72] Mortier E, Woo T, Advincula R, Gozalo S, Ma A (2008). IL-15Ralpha chaperones IL-15 to stable dendritic cell membrane complexes that activate NK cells via trans presentation. J Exp Med.

[73] Barr DP, Belz GT, Reading PC, Wojtasiak M, Whitney PG, Heath WR, Carbone FR, Brooks AG (2007). A role for plasmacytoid dendritic cells in the rapid IL-18-dependent activation of NK cells following HSV-1 infection. Eur J Immunol.

[74] Decalf J, Fernandes S, Longman R, Ahloulay M, Audat F, Lefrerre F, Rice CM, Pol S, Albert ML (2007). Plasmacytoid dendritic cells initiate a complex chemokine and cytokine network and are a viable drug target in chronic HCV patients. J Exp Med.

[75] Piqueras B, Connolly J, Freitas H, Palucka AK, Banchereau J (2006). Upon viral exposure, myeloid and plasmacytoid dendritic cells produce 3 waves of distinct chemokines to recruit immune effectors. Blood.

[76] Hanabuchi S, Watanabe N, Wang YH, Ito T, Shaw J, Cao W, Qin FX, Liu YJ (2006). Human plasmacytoid predendritic cells activate NK cells through glucocorticoid-induced tumor necrosis factor receptor-ligand (GITRL). Blood.

[77] Pichlmair A, Reis e Sousa C (2007). Innate recognition of viruses. Immunity.

[78] Cervantes-Barragan L, Zust R, Weber F, Spiegel M, Lang KS, Akira S, Thiel V, Ludewig B (2007). Control of coronavirus infection through plasmacytoid dendritic-cell-derived type I interferon. Blood.

[79] Cervantes-Barragan L, Kalinke U, Zust R, Konig M, Reizis B, Lopez-Macias C, Thiel V, Ludewig B (2009). Type I IFN-mediated protection of macrophages and dendritic cells secures control of murine coronavirus infection. J Immunol.

[80] Crozat K, Vivier E, Dalod M (2009). Crosstalk between components of the innate immune system: promoting anti-microbial defenses and avoiding immunopathologies. Immunol Rev.

[81] Barton GM, Kagan JC, Medzhitov R (2006). Intracellular localization of Toll-like receptor 9 prevents recognition of self DNA but facilitates access to viral DNA. Nat Immunol.

[82] Megjugorac NJ, Jacobs ES, Izaguirre AG, George TC, Gupta G, Fitzgerald-Bocarsly P (2007). Image-based study of interferongenic interactions between plasmacytoid dendritic cells and HSV-infected monocyte-derived dendritic cells. Immunol Invest.

[83] Lund J, Sato A, Akira S, Medzhitov R, Iwasaki A (2003). Toll-like receptor 9-mediated recognition of Herpes simplex virus-2 by plasmacytoid dendritic cells. J Exp Med.

[84] Delale T, Paquin A, Asselin-Paturel C, Dalod M, Brizard G, Bates EE, Kastner P, Chan S, Akira S, Vicari A, Biron CA, Trinchieri G, Briere F (2005). MyD88-dependent and -independent murine cytomegalovirus sensing for IFN-alpha release and initiation of immune responses in vivo. J Immunol.

[85] Krug A, Luker GD, Barchet W, Leib DA, Akira S, Colonna M (2004). Herpes simplex virus type 1 activates murine natural interferon-producing cells through toll-like receptor 9. Blood.

[86] Schneider K, Loewendorf A, De Trez C, Fulton J, Rhode A, Shumway H, Ha S, Patterson G, Pfeffer K, Nedospasov SA, Ware CF, Benedict CA (2008). Lymphotoxin-mediated crosstalk between B cells and splenic stroma promotes the initial type I interferon response to cytomegalovirus. Cell Host Microbe.

[87] Rebsamen M, Heinz LX, Meylan E, Michallet MC, Schroder K, Hofmann K, Vazquez J, Benedict CA, Tschopp J (2009). DAI/ZBP1 recruits RIP1 and RIP3 through RIP homotypic interaction motifs to activate NF-kappaB. EMBO Rep.

[88] Samanta M, Iwakiri D, Kanda T, Imaizumi T, Takada K (2006). EB virus-encoded RNAs are recognized by RIG-I and activate signaling to induce type I IFN. EMBO J.

[89] Zucchini N, Bessou G, Traub S, Robbins SH, Uematsu S, Akira S, Alexopoulou L, Dalod M (2008). Cutting edge: Overlapping functions of TLR7 and TLR9 for innate defense against a herpesvirus infection. J Immunol.

[90] Mancuso G, Gambuzza M, Midiri A, Biondo C, Papasergi S, Akira S, Teti G, Beninati C (2009). Bacterial recognition by TLR7 in the lysosomes of conventional dendritic cells. Nat Immunol.

[91] Fukui R, Saitoh S, Matsumoto F, Kozuka-Hata H, Oyama M, Tabeta K, Beutler B, Miyake K (2009). Unc93B1 biases Toll-like receptor responses to nucleic acid in dendritic cells toward DNA- but against RNA-sensing. J Exp Med.

[92] Honda K, Yanai H, Negishi H, Asagiri M, Sato M, Mizutani T, Shimada N, Ohba Y, Takaoka A, Yoshida N, Taniguchi T (2005). IRF-7 is the master regulator of type-I interferon-dependent immune responses. Nature.

[93] Levy DE, Marie I, Smith E, Prakash A (2002). Enhancement and diversification of IFN induction by IRF-7-mediated positive feedback. J Interferon Cytokine Res.

[94] Guiducci C, Ott G, Chan JH, Damon E, Calacsan C, Matray T, Lee KD, Coffman RL, Barrat FJ (2006). Properties regulating the nature of the plasmacytoid dendritic cell response to Toll-like receptor 9 activation. J Exp Med.

[95] Honda K, Ohba Y, Yanai H, Negishi H, Mizutani T, Takaoka A, Taya C, Taniguchi T (2005). Spatiotemporal regulation of MyD88-IRF-7 signalling for robust type-I interferon induction. Nature.

[96] Barchet W, Cella M, Odermatt B, Asselin-Paturel C, Colonna M, Kalinke U (2002). Virus-induced interferon alpha production by a dendritic cell subset in the absence of feedback signaling in vivo. J Exp Med.

[97] Kumagai Y, Kumar H, Koyama S, Kawai T, Takeuchi O, Akira S (2009). Cutting Edge: TLR-Dependent viral recognition along with type I IFN positive feedback signaling masks the requirement of viral replication for IFN-{alpha} production in plasmacytoid dendritic cells. J Immunol.

[98] Diebold SS, Montoya M, Unger H, Alexopoulou L, Roy P, Haswell LE, Al-Shamkhani A, Flavell R, Borrow P, Reis e Sousa C (2003). Viral infection switches non-plasmacytoid dendritic cells into high interferon producers. Nature.

[99] DeFilippis VR (2007). Induction and evasion of the type I interferon response by cytomegaloviruses. Adv Exp Med Biol.

[100] Hengel H, Koszinowski UH, Conzelmann KK (2005). Viruses know it all: new insights into IFN networks. Trends Immunol.

[101] Bowie AG, Unterholzner L (2008). Viral evasion and subversion of pattern-recognition receptor signalling. Nat Rev Immunol.

[102] Chang WL, Barry PA, Szubin R, Wang D, Baumgarth N (2009). Human cytomegalovirus suppresses type I interferon secretion by plasmacytoid dendritic cells through its interleukin 10 homolog. Virology.

[103] Banchereau J, Pascual V (2006). Type I interferon in systemic lupus erythematosus and other autoimmune diseases. Immunity.

[104] Conrad C, Meller S, Gilliet M (2009). Plasmacytoid dendritic cells in the skin: to sense or not to sense nucleic acids. Semin Immunol.

[105] Nestle FO, Conrad C, Tun-Kyi A, Homey B, Gombert M, Boyman O, Burg G, Liu YJ, Gilliet M (2005). Plasmacytoid predendritic cells initiate psoriasis through interferon-alpha production. J Exp Med.

[106] Cao W, Rosen DB, Ito T, Bover L, Bao M, Watanabe G, Yao Z, Zhang L, Lanier LL, Liu YJ (2006). Plasmacytoid dendritic cell-specific receptor ILT7-Fc epsilonRI gamma inhibits Toll-like receptor-induced interferon production. J Exp Med.

[107] Rock J, Schneider E, Grun JR, Grutzkau A, Kuppers R, Schmitz J, Winkels G (2007). CD303 (BDCA-2) signals in plasmacytoid dendritic cells via a BCR-like signalosome involving Syk, Slp65 and PLCgamma2. Eur J Immunol.

[108] Cao W, Zhang L, Rosen DB, Bover L, Watanabe G, Bao M, Lanier LL, Liu YJ (2007). BDCA2/Fc epsilon RI gamma complex signals through a novel BCR-like pathway in human plasmacytoid dendritic cells. PLoS Biol.

[109] Blasius A, Vermi W, Krug A, Facchetti F, Cella M, Colonna M (2004). A cell-surface molecule selectively expressed on murine natural interferon-producing cells that blocks secretion of interferon-alpha. Blood.

[110] Blasius AL, Cella M, Maldonado J, Takai T, Colonna M (2006). Siglec-H is an IPC-specific receptor that modulates type I IFN secretion through DAP12. Blood.

[111] Sjolin H, Robbins SH, Bessou G, Hidmark A, Tomasello E, Johansson M, Hall H, Charifi F, Karlsson Hedestam GB, Biron CA, Karre K, Hoglund P, Vivier E, Dalod M (2006). DAP12 signaling regulates plasmacytoid dendritic cell homeostasis and down-modulates their function during viral infection. J Immunol.

[112] Blasius AL, Giurisato E, Cella M, Schreiber RD, Shaw AS, Colonna M (2006). Bone marrow stromal cell antigen 2 is a specific marker of type I IFN-producing cells in the naive mouse, but a promiscuous cell surface antigen following IFN stimulation. J Immunol.

[113] Rothlin CV, Ghosh S, Zuniga EI, Oldstone MB, Lemke G (2007). TAM receptors are pleiotropic inhibitors of the innate immune response. Cell.

[114] Meyer-Wentrup F, Benitez-Ribas D, Tacken PJ, Punt CJ, Figdor CG, de Vries IJ, Adema GJ (2008). Targeting DCIR on human plasmacytoid dendritic cells results in antigen presentation and inhibits IFN-alpha production. Blood.

[115] Zhang J, Raper A, Sugita N, Hingorani R, Salio M, Palmowski MJ, Cerundolo V, Crocker PR (2006). Characterization of Siglec-H as a novel endocytic receptor expressed on murine plasmacytoid dendritic cell precursors. Blood.

[116] Robbins SH, Bessou G, Cornillon A, Zucchini N, Rupp B, Ruzsics Z, Sacher T, Tomasello E, Vivier E, Koszinowski UH, Dalod M (2007). Natural killer cells promote early CD8 T cell responses against cytomegalovirus. PLoS Pathog.

[117] Villadangos JA, Young L (2008). Antigen-presentation properties of plasmacytoid dendritic cells. Immunity.

[118] Young LJ, Wilson NS, Schnorrer P, Proietto A, ten Broeke T, Matsuki Y, Mount AM, Belz GT, O’Keeffe M, Ohmura-Hoshino M, Ishido S, Stoorvogel W, Heath WR, Shortman K, Villadangos JA (2008). Differential MHC class II synthesis and ubiquitination confers distinct antigen-presenting properties on conventional and plasmacytoid dendritic cells. Nat Immunol.

[119] Sadaka C, Marloie-Provost MA, Soumelis V, Benaroch P (2009). Developmental regulation of MHC II expression and transport in human plasmacytoid-derived dendritic cells. Blood.

[120] Hoeffel G, Ripoche AC, Matheoud D, Nascimbeni M, Escriou N, Lebon P, Heshmati F, Guillet JG, Gannage M, Caillat-Zucman S, Casartelli N, Schwartz O, De la Salle H, Hanau D, Hosmalin A, Maranon C (2007). Antigen crosspresentation by human plasmacytoid dendritic cells. Immunity.

[121] Mouries J, Moron G, Schlecht G, Escriou N, Dadaglio G, Leclerc C (2008). Plasmacytoid dendritic cells efficiently cross-prime naive T cells in vivo after TLR activation. Blood.

[122] Di Pucchio T, Chatterjee B, Smed-Sorensen A, Clayton S, Palazzo A, Montes M, Xue Y, Mellman I, Banchereau J, Connolly JE (2008). Direct proteasome-independent cross-presentation of viral antigen by plasmacytoid dendritic cells on major histocompatibility complex class I. Nat Immunol.

[123] Yoneyama H, Matsuno K, Toda E, Nishiwaki T, Matsuo N, Nakano A, Narumi S, Lu B, Gerard C, Ishikawa S, Matsushima K (2005). Plasmacytoid DCs help lymph node DCs to induce anti-HSV CTLs. J Exp Med.

[124] Yoneyama H, Matsuno K, Zhang Y, Nishiwaki T, Kitabatake M, Ueha S, Narumi S, Morikawa S, Ezaki T, Lu B, Gerard C, Ishikawa S, Matsushima K (2004). Evidence for recruitment of plasmacytoid dendritic cell precursors to inflamed lymph nodes through high endothelial venules. Int Immunol.

[125] Jego G, Palucka AK, Blanck JP, Chalouni C, Pascual V, Banchereau J (2003). Plasmacytoid dendritic cells induce plasma cell differentiation through type I interferon and interleukin 6. Immunity.

[126] Kawamura K, Kadowaki N, Kitawaki T, Uchiyama T (2006). Virus-stimulated plasmacytoid dendritic cells induce CD4+ cytotoxic regulatory T cells. Blood.

[127] Kvale EO, Floisand Y, Lund-Johansen F, Rollag H, Farkas L, Ghanekar S, Brandtzaeg P, Jahnsen FL, Olweus J (2007). Plasmacytoid DCs regulate recall responses by rapid induction of IL-10 in memory T cells. Blood.

[128] Fallarino F, Asselin-Paturel C, Vacca C, Bianchi R, Gizzi S, Fioretti MC, Trinchieri G, Grohmann U, Puccetti P (2004). Murine plasmacytoid dendritic cells initiate the immunosuppressive pathway of tryptophan catabolism in response to CD200 receptor engagement. J Immunol.

[129] Mellor AL, Baban B, Chandler PR, Manlapat A, Kahler DJ, Munn DH (2005). Cutting edge: CpG oligonucleotides induce splenic CD19+ dendritic cells to acquire potent indoleamine 2, 3-dioxygenase-dependent T cell regulatory functions via IFN Type 1 signaling. J Immunol.

[130] Sharma MD, Baban B, Chandler P, Hou DY, Singh N, Yagita H, Azuma M, Blazar BR, Mellor AL, Munn DH (2007). Plasmacytoid dendritic cells from mouse tumor-draining lymph nodes directly activate mature Tregs via indoleamine 2, 3-dioxygenase. J Clin Invest.

[131] Boasso A, Herbeuval JP, Hardy AW, Anderson SA, Dolan MJ, Fuchs D, Shearer GM (2007). HIV inhibits CD4+ T-cell proliferation by inducing indoleamine 2, 3-dioxygenase in plasmacytoid dendritic cells. Blood.

[132] Manches O, Munn D, Fallahi A, Lifson J, Chaperot L, Plumas J, Bhardwaj N (2008). HIV-activated human plasmacytoid DCs induce Tregs through an indoleamine 2, 3-dioxygenase-dependent mechanism. J Clin Invest.

[133] Rolle A, Olweus J (2009). Dendritic cells in cytomegalovirus infection: viral evasion and host countermeasures. APMIS.

[134] Lund JM, Hsing L, Pham TT, Rudensky AY (2008). Coordination of early protective immunity to viral infection by regulatory T cells. Science.

[135] Ronnblom L, Eloranta ML, Alm GV (2003). Role of natural interferon-alpha producing cells (plasmacytoid dendritic cells) in autoimmunity. Autoimmunity.

[136] Stacey AR, Norris PJ, Qin L, Haygreen EA, Taylor E, Heitman J, Lebedeva M, DeCamp A, Li D, Grove D, Self SG, Borrow P (2009). Induction of a striking systemic cytokine cascade prior to peak viremia in acute human immunodeficiency virus type 1 infection, in contrast to more modest and delayed responses in acute hepatitis B and C virus infections. J Virol.

[137] Hyrcza MD, Kovacs C, Loutfy M, Halpenny R, Heisler L, Yang S, Wilkins O, Ostrowski M, Der SD (2007). Distinct transcriptional profiles in ex vivo CD4+ and CD8+ T cells are established early in human immunodeficiency virus type 1 infection and are characterized by a chronic interferon response as well as extensive transcriptional changes in CD8+ T cells. J Virol.

[138] Mandl JN, Barry AP, Vanderford TH, Kozyr N, Chavan R, Klucking S, Barrat FJ, Coffman RL, Staprans SI, Feinberg MB (2008). Divergent TLR7 and TLR9 signaling and type I interferon production distinguish pathogenic and nonpathogenic AIDS virus infections. Nat Med.

[139] Boasso A, Shearer GM, Chougnet C (2009). Immune dysregulation in human immunodeficiency virus infection: know it, fix it, prevent it. J Intern Med.

[140] Lehmann C, Harper JM, Taubert D, Hartmann P, Fatkenheuer G, Jung N, van Lunzen J, Stellbrink HJ, Gallo RC, Romerio F (2008). Increased interferon alpha expression in circulating plasmacytoid dendritic cells of HIV-1-infected patients. J Acquir Immune Defic Syndr.

[141] Meier A, Chang JJ, Chan ES, Pollard RB, Sidhu HK, Kulkarni S, Wen TF, Lindsay RJ, Orellana L, Mildvan D, Bazner S, Streeck H, Alter G, Lifson JD, Carrington M, Bosch RJ, Robbins GK, Altfeld M (2009). Sex differences in the Toll-like receptor-mediated response of plasmacytoid dendritic cells to HIV-1. Nat Med.

[142] Diop OM, Ploquin MJ, Mortara L, Faye A, Jacquelin B, Kunkel D, Lebon P, Butor C, Hosmalin A, Barre-Sinoussi F, Muller-Trutwin MC (2008). Plasmacytoid dendritic cell dynamics and alpha interferon production during Simian immunodeficiency virus infection with a nonpathogenic outcome. J Virol.

[143] Riviere Y, Gresser I, Guillon JC, Tovey MG (1977). Inhibition by anti-interferon serum of lymphocytic choriomeningitis virus disease in suckling mice. Proc Natl Acad Sci U S A.

[144] Yen JH, Ganea D (2009). Interferon beta induces mature dendritic cell apoptosis through caspase-11 / caspase-3 activation. Blood.

[145] Hahm B, Trifilo MJ, Zuniga EI, Oldstone MB (2005). Viruses evade the immune system through type I interferon-mediated STAT2-dependent, but STAT1-independent, signaling. Immunity.

[146] Binder D, Fehr J, Hengartner H, Zinkernagel RM (1997). Virus-induced transient bone marrow aplasia: major role of interferon-alpha/beta during acute infection with the noncytopathic lymphocytic choriomeningitis virus. J Exp Med.

[147] Lin Q, Dong C, Cooper MD (1998). Impairment of T and B cell development by treatment with a type I interferon. J Exp Med.

[148] Bahl K, Kim SK, Calcagno C, Ghersi D, Puzone R, Celada F, Selin LK, Welsh RM (2006). IFN-induced attrition of CD8 T cells in the presence or absence of cognate antigen during the early stages of viral infections. J Immunol.

[149] Gil MP, Salomon R, Louten J, Biron CA (2006). Modulation of STAT1 protein levels: a mechanism shaping CD8 T-cell responses in vivo. Blood.

[150] Le Bon A, Schiavoni G, D’Agostino G, Gresser I, Belardelli F, Tough DF (2001). Type i interferons potently enhance humoral immunity and can promote isotype switching by stimulating dendritic cells in vivo. Immunity.

[151] Le Bon A, Thompson C, Kamphuis E, Durand V, Rossmann C, Kalinke U, Tough DF (2006). Cutting edge: enhancement of antibody responses through direct stimulation of B and T cells by type I IFN. J Immunol.

[152] Navarini AA, Recher M, Lang KS, Georgiev P, Meury S, Bergthaler A, Flatz L, Bille J, Landmann R, Odermatt B, Hengartner H, Zinkernagel RM (2006). Increased susceptibility to bacterial superinfection as a consequence of innate antiviral responses. Proc Natl Acad Sci U S A.

[153] Shahangian A, Chow EK, Tian X, Kang JR, Ghaffari A, Liu SY, Belperio JA, Cheng G, Deng JC (2009). Type I IFNs mediate development of postinfluenza bacterial pneumonia in mice. J Clin Invest.

[154] Iversen AC, Steinkjer B, Nilsen N, Bohnhorst J, Moen SH, Vik R, Stephens P, Thomas DW, Benedict CA, Espevik T (2009). A proviral role for CpG in cytomegalovirus infection. J Immunol.

[155] Li Q, Skinner PJ, Ha SJ, Duan L, Mattila TL, Hage A, White C, Barber DL, O’Mara L, Southern PJ, Reilly CS, Carlis JV, Miller CJ, Ahmed R, Haase AT (2009). Visualizing antigen-specific and infected cells in situ predicts outcomes in early viral infection. Science.

[156] Orange JS, Salazar-Mather TP, Opal SM, Biron CA (1997). Mechanisms for virus-induced liver disease: tumor necrosis factor-mediated pathology independent of natural killer and T cells during murine cytomegalovirus infection. J Virol.

[157] Banchereau J, Pascual V, Palucka AK (2004). Autoimmunity through cytokine-induced dendritic cell activation. Immunity.

[158] Harley JB, Harley IT, Guthridge JM, James JA (2006). The curiously suspicious: a role for Epstein-Barr virus in lupus. Lupus.

[159] Posnett DN (2008). Herpesviruses and autoimmunity. Curr Opin Investig Drugs.

[160] Shen H, Iwasaki A (2006). A crucial role for plasmacytoid dendritic cells in antiviral protection by CpG ODN-based vaginal microbicide. J Clin Invest.

[161] Martinez V, Molina JM, Scieux C, Ribaud P, Morfin F (2006). Topical imiquimod for recurrent acyclovir-resistant HSV infection. Am J Med.

[162] Vollstedt S, Franchini M, Hefti HP, Odermatt B, O’Keeffe M, Alber G, Glanzmann B, Riesen M, Ackermann M, Suter M (2003). Flt3 ligand-treated neonatal mice have increased innate immunity against intracellular pathogens and efficiently control virus infections. J Exp Med.

[163] Li Q, Estes JD, Schlievert PM, Duan L, Brosnahan AJ, Southern PJ, Reilly CS, Peterson ML, Schultz-Darken N, Brunner KG, Nephew KR, Pambuccian S, Lifson JD, Carlis JV, Haase AT (2009). Glycerol monolaurate prevents mucosal SIV transmission. Nature.

[164] Guiducci C, Coffman RL, Barrat FJ (2009). Signalling pathways leading to IFN-alpha production in human plasmacytoid dendritic cell and the possible use of agonists or antagonists of TLR7 and TLR9 in clinical indications. J Intern Med.

[165] Aldridge JR, Moseley CE, Boltz DA, Negovetich NJ, Reynolds C, Franks J, Brown SA, Doherty PC, Webster RG, Thomas PG (2009). TNF/iNOS-producing dendritic cells are the necessary evil of lethal influenza virus infection. Proc Natl Acad Sci U S A.

[166] Querec T, Bennouna S, Alkan S, Laouar Y, Gorden K, Flavell R, Akira S, Ahmed R, Pulendran B (2006). Yellow fever vaccine YF-17D activates multiple dendritic cell subsets via TLR2, 7, 8, and 9 to stimulate polyvalent immunity. J Exp Med.

[167] Karrer U, Sierro S, Wagner M, Oxenius A, Hengel H, Koszinowski UH, Phillips RE, Klenerman P (2003). Memory inflation: continuous accumulation of antiviral CD8+ T cells over time. J Immunol.

[168] Simon CO, Holtappels R, Tervo HM, Bohm V, Daubner T, Oehrlein-Karpi SA, Kuhnapfel B, Renzaho A, Strand D, Podlech J, Reddehase MJ, Grzimek NK (2006). CD8 T cells control cytomegalovirus latency by epitope-specific sensing of transcriptional reactivation. J Virol.

[169] Karrer U, Wagner M, Sierro S, Oxenius A, Hengel H, Dumrese T, Freigang S, Koszinowski UH, Phillips RE, Klenerman P (2004). Expansion of protective CD8+ T-cell responses driven by recombinant cytomegaloviruses. J Virol.

[170] Hansen SG, Vieville C, Whizin N, Coyne-Johnson L, Siess DC, Drummond DD, Legasse AW, Axthelm MK, Oswald K, Trubey CM, Piatak M, Lifson JD, Nelson JA, Jarvis MA, Picker LJ (2009). Effector memory T cell responses are associated with protection of rhesus monkeys from mucosal simian immunodeficiency virus challenge. Nat Med.

[171] Casrouge A, Zhang SY, Eidenschenk C, Jouanguy E, Puel A, Yang K, Alcais A, Picard C, Mahfoufi N, Nicolas N, Lorenzo L, Plancoulaine S, Senechal B, Geissmann F, Tabeta K, Hoebe K, Du X, Miller RL, Heron B, Mignot C, de Villemeur TB, Lebon P, Dulac O, Rozenberg F, Beutler B, Tardieu M, Abel L, Casanova JL (2006). Herpes simplex virus encephalitis in human UNC-93B deficiency. Science.

[172] Yang K, Puel A, Zhang S, Eidenschenk C, Ku CL, Casrouge A, Picard C, von Bernuth H, Senechal B, Plancoulaine S, Al-Hajjar S, Al-Ghonaium A, Marodi L, Davidson D, Speert D, Roifman C, Garty BZ, Ozinsky A, Barrat FJ, Coffman RL, Miller RL, Li X, Lebon P, Rodriguez-Gallego C, Chapel H, Geissmann F, Jouanguy E, Casanova JL (2005). Human TLR-7-, -8-, and -9-mediated induction of IFN-alpha/beta and -lambda Is IRAK-4 dependent and redundant for protective immunity to viruses. Immunity.

[173] Barreiro LB, Ben-Ali M, Quach H, Laval G, Patin E, Pickrell JK, Bouchier C, Tichit M, Neyrolles O, Gicquel B, Kidd JR, Kidd KK, Alcais A, Ragimbeau J, Pellegrini S, Abel L, Casanova JL, Quintana-Murci L (2009). Evolutionary dynamics of human Toll-like receptors and their different contributions to host defense. PLoS Genet.

